# Network resonance can be generated independently at distinct levels of neuronal organization

**DOI:** 10.1371/journal.pcbi.1010364

**Published:** 2022-07-18

**Authors:** Eran Stark, Amir Levi, Horacio G. Rotstein

**Affiliations:** 1 Sagol School of Neuroscience and Department of Physiology and Pharmacology, Sackler Faculty of Medicine, Tel Aviv University, Tel Aviv, Israel; 2 Federated Department of Biological Sciences, New Jersey Institute of Technology and Rutgers University, Newark, New Jersey, United States of America; University of Pittsburgh, UNITED STATES

## Abstract

Resonance is defined as maximal response of a system to periodic inputs in a limited frequency band. Resonance may serve to optimize inter-neuronal communication, and has been observed at multiple levels of neuronal organization. However, it is unknown how neuronal resonance observed at the network level is generated and how network resonance depends on the properties of the network building blocks. Here, we first develop a metric for quantifying spike timing resonance in the presence of background noise, extending the notion of spiking resonance for in vivo experiments. Using conductance-based models, we find that network resonance can be inherited from resonances at other levels of organization, or be intrinsically generated by combining mechanisms across distinct levels. Resonance of membrane potential fluctuations, postsynaptic potentials, and single neuron spiking can each be generated independently of resonance at any other level and be propagated to the network level. At all levels of organization, interactions between processes that give rise to low- and high-pass filters generate the observed resonance. Intrinsic network resonance can be generated by the combination of filters belonging to different levels of organization. Inhibition-induced network resonance can emerge by inheritance from resonance of membrane potential fluctuations, and be sharpened by presynaptic high-pass filtering. Our results demonstrate a multiplicity of qualitatively different mechanisms that can generate resonance in neuronal systems, and provide analysis tools and a conceptual framework for the mechanistic investigation of network resonance in terms of circuit components, across levels of neuronal organization.

## Author summary

How one part of the brain responds to periodic input from another part depends on resonant circuit properties. Resonance is a basic property of physical systems, and has been experimentally observed at various levels of neuronal organization both in vitro and in vivo. Yet how resonance is generated in neuronal networks is largely unknown. In particular, whether resonance can be generated directly at the level of a network of spiking neurons remains to be determined. Using detailed biophysical modeling, we develop a conceptual framework according to which resonance at a given level of organization is generated by the interplay of low- and high-pass filters, implemented at either the same or across levels of neuronal organization. We tease apart representative, biophysically-plausible generative mechanisms of resonance at four different levels of organization: membrane potential fluctuations, single neuron spiking, synaptic transmission, and neuronal networks. We identify conditions under which resonance at one level can be inherited to another level of organization, provide conclusive evidence that resonance at each level can be generated without resonance at any other level, and describe a number of representative routes to network resonance. The proposed framework facilitates the investigation of resonance in neuronal systems.

## Introduction

Resonance refers to the maximal response of a system to periodic input in a limited (finite non-zero; “resonant”) frequency band. In neuronal systems, resonance has been observed at multiple levels of organization and quantified using various metrics, in all cases capturing the notion of optimal gain. In the simplest case, similarly to RLC circuits, the subthreshold response of an isolated neuron to oscillatory inputs has been measured in terms of the impedance amplitude profile, quantifying the amplitude response of the membrane potential fluctuations as a function of the input frequency [1–7]. A neuron exhibits cellular-level resonance of membrane potential fluctuations if the impedance magnitude peaks at a non-zero frequency. Otherwise, individual neurons may behave as low-pass filters [6,8,9] or may exhibit more complex behavior depending on the number and type of ionic currents and their time scales [8,10–12]. In addition to resonance of membrane potential fluctuations, cellular-level resonance may occur at the spiking level: spikes may preferentially occur at specific frequencies of an oscillatory input current [2,8], yielding spiking resonance. Beyond the cellular level, resonance may occur at the level of synaptic transmission: the amplitude of postsynaptic potentials (PSPs) may peak at some instantaneous rate of the presynaptic spikes [13–15]. Finally, computational modeling [16–21] and in vivo experiments [22] showed that resonance may occur at the network level.

Theoretical studies have shown that subthreshold resonance can be communicated to the spiking regime [11,23,24]. A possible implication of this observation is that resonance can be inherited over levels of neuronal organization, either directly or indirectly. For instance, subthreshold resonance at theta frequencies may be expected to create spiking resonance at theta frequencies, which may in turn generate network resonance at theta frequencies when resonant spiking neurons interact with other neurons. Alternatively, the interplay of the positive and slower negative feedback effects operating at interacting levels of organization may communicate resonance across these levels. However, direct periodic activation of hippocampal CA1 pyramidal cells that have been shown to exhibit subthreshold resonance in vitro [3,25] did not produce network resonance in vivo, whereas direct activation of inhibitory neurons did [22]. Thus, it is still unclear whether and under what conditions resonance at one level of organization is causally related to (e.g., is inherited from) resonance at another level. One obstacle to addressing these issues is the lack of a general framework for investigating the mechanisms of generation of neuronal resonance in terms of the frequency-preference properties of system components.

The specific question we address in this paper is whether resonance observed at one level of organization is necessarily inherited from resonance at lower levels of organization (e.g., membrane potential fluctuations, single neuron spiking, postsynaptic potentials). Previous work showed the presence of resonance in networks of rate models [19,20]. Other work demonstrated resonance in spiking neurons [23,26–29]. However, a direct link between resonance in a single spiking neuron and a network of spiking neurons has not been shown (although see [19], describing a comparative analysis between resonance in networks of spiking neurons and rate mdoels). An alternative manner in which network resonance can be generated is by the existence of independent processes that may share some building blocks, and act to generate resonance at distinct levels. This alternative scenario does not preclude the existence of neuronal systems in which resonances are communicated across levels of organization, particularly from the subthreshold to the network levels.

To tackle this question, we carry out detailed conductance-based modeling of individual neurons and neuronal networks. We identify and analyze a number of case studies at various levels of organization and increasing levels of complexity, where the generation of resonance depends on mechanisms confined to each level. Capturing the complexity of the problem, particularly the interaction between levels of organization, requires going beyond the linear domain and weak signals where the classical mathematical analysis of linear systems is possible and mean-field theory of irregularly spiking neurons is applicable. Therefore we entirely rely on computer simulation of a number of scenarios carefully designed to address a specific question or shed light on a specific issue. We find that despite the nonlinearities and complexity of the neuronal systems examined, the resonance-generating mechanisms can be described in terms of the interplay of low-pass filters (LPFs) and high-pass filters (HPFs). The filtering building blocks (or modules) depend on the biophysical and dynamic details and structure specific to each level. In contrast, network resonance can be generated by combining low- and high-pass filtering mechanisms across levels of organization, in the lack of resonance at any other level of organization.

## Results

### Two distinct types of spiking resonance: cycle-averaged firing rate resonance and spike timing resonance

**Fig 1:**
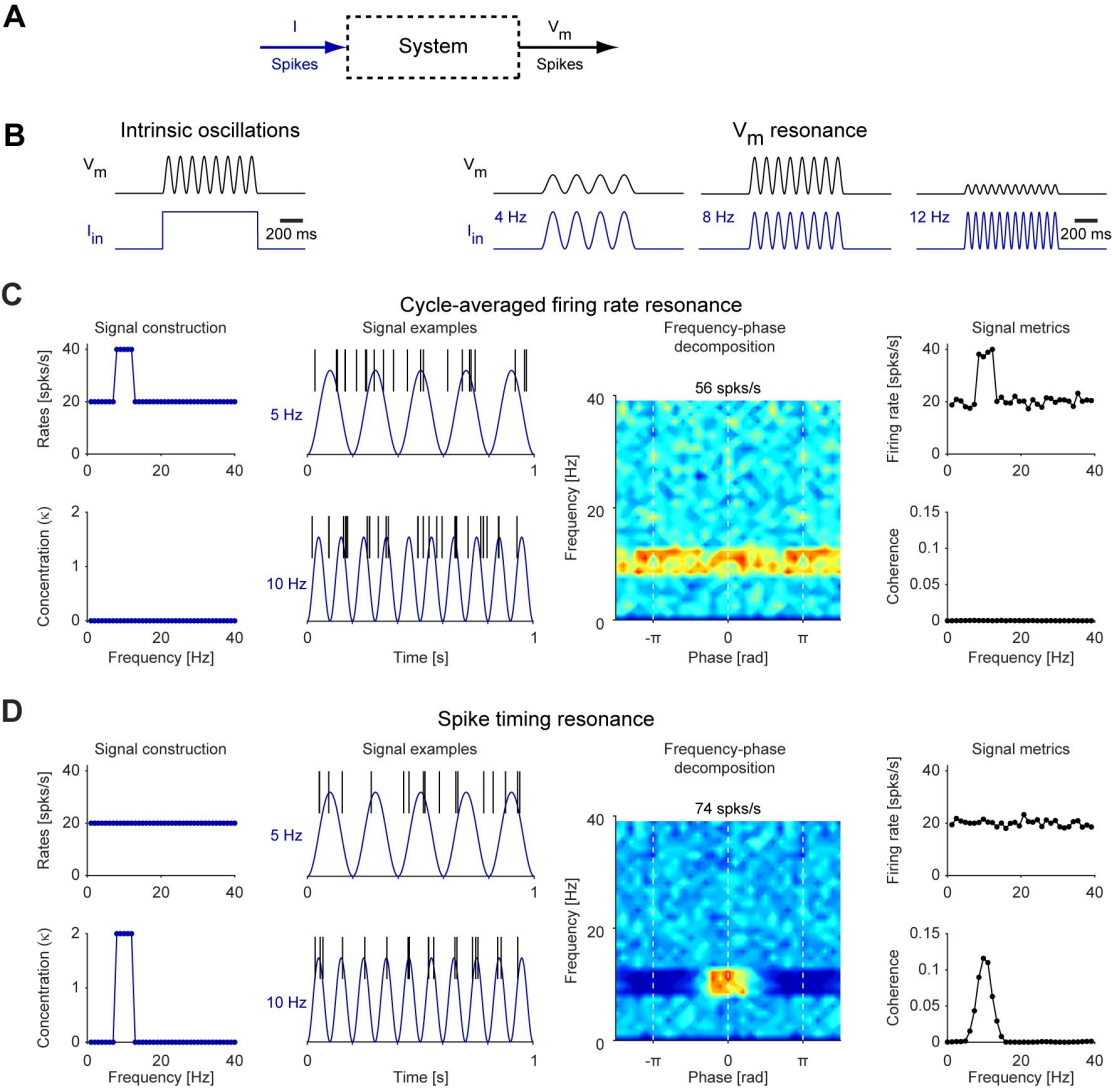
Cycle-averaged firing rate resonance and spike timing resonance. (**A**) To quantify the response, a system is given an input (e.g., current or spikes) and the output is measured. (**B**) *Left*: Induced oscillations are defined are as a rhythmic output in response to a non-rhythmic (e.g., pulse or noise) input. *Right*: Resonance is defined as a maximal response of the system to periodic input at a non-zero finite input frequency or frequency band. In neuronal systems, this definition readily applies to analog quantities, e.g., the membrane potential fluctuations. (**C**) Cycle-averaged firing rate resonance is a direct extension of the analog quantity. A synthetic neuronal signal was constructed in which firing rate at the 8–12 Hz range was twice the firing rate at other frequencies (*top left*). Actual spike trains were realized by randomly drawing the number of spikes per cycle from a Poisson distribution. This corresponds to a horizontal band in the fingerprint, a 2D frequency-phase map of instantaneous firing rates (*second panel from right*). Here and in all fingerprints, blue corresponds to 0 spk/s, and red corresponds to the instantaneous firing rate indicated in the title (here, 56 spk/s). The image is expanded to show 1½ cycles in the phase axis (abscissa). In this configuration, resonance is fully quantified by the cycle-averaged firing rate (*top right*). (**D**) In spike timing resonance, the firing rate may be identical at all input frequencies (*top left*), but spikes occur at specific phases in the resonant frequency band. A signal was constructed in which the phase of every spike was drawn randomly from a von Mises distribution, for which the concentration parameter κ was higher at the 8–12 Hz range (*bottom left*). This corresponds to a high instantaneous firing rate at a specific combination of frequency and phase (red patch in the fingerprint; *second panel from right*). In this configuration, the cycle-averaged firing rates are similar across frequencies (*top right*), and resonance can be quantified using the input-output spectral coherence metric (*bottom right*).

In the context of rhythmic systems (**Fig 1A**), one can differentiate between two types of responses: an oscillator and a resonator. In an electric oscillator that receives as input a square pulse of current, the output is an oscillatory voltage (**Fig 1B, left**). The generation of oscillations in neuronal systems has been studied extensively [7,30]. A second type of rhythmic system is a resonator (**Fig 1B, right**). Resonance is defined as a maximal response of the system to a periodic input at a non-zero finite frequency or frequency band. In neuronal systems, resonance has often been discussed in the context of current input to a single neuron [31]. In a single neuron, resonance at the subthreshold level occurs when the amplitude of the response variable (e.g., voltage: the membrane potential, *V*_*m*_) peaks at a non-zero frequency of the input (e.g., current) applied to the neuron (**Fig 1B, right**). This can be quantified using the impedance amplitude profile, capturing the ratio between the output and input amplitudes at every input frequency. Ultimately, neurons transmit their output as spikes. A natural direct extension of the analog (subthreshold) definition of resonance to the spiking domain is “cycle-averaged firing rate resonance” (**Fig 1C**), which can be fully quantified by the cycle-averaged firing rate metric. In cycle-averaged firing rate resonance, the rate of spikes fired by the neuron is maximal when the frequency of the input (e.g., the presynaptic spike train or the current applied to the neuron) is at a non-zero frequency band.

The usage of a discrete output (spikes) allows a second type of resonance to be considered, which we denote as “spike timing resonance” (**Fig 1D**). In spike timing resonance, the cycle-averaged firing rate can be the same for all input frequencies (**Fig 1D, top left**). However, spikes occur at a more limited range of phases at some frequency (e.g., 10 Hz; **Fig 1D, bottom left**) compared to other frequencies (e.g., 5 or 15 Hz; **Fig 1D, bottom left**). Hence the output, namely the instantaneous firing rate, is maximal at a given phase of a non-zero finite frequency (the resonant frequency). Therefore, spike phase must be taken into account when quantifying the preferred frequency response phenomenon. In this setting, the input (i.e., the oscillatory current) and the output (i.e., the spike times) are more coherent at the resonant frequencies (**Fig 1D, bottom right**). The spikes exhibit more consistent phase locking at the resonant frequencies, which can be quantified using the spectral coherence. For the remainder of this article, we refer to the magnitude of the complex spectral coherence simply as “coherence”. Coherence ranges 0–1 and is maximal when spikes exhibit perfect phase locking to the periodic input. Thus, in spike timing resonance, the coherence metric exhibits a maximum at a finite, non-zero frequency.

In principle (and as illustrated in **Fig 1CD**), cycle-averaged firing rate resonance and spike timing resonance are independent phenomena, and one can occur without the other. Indeed, previous work in freely-moving mice showed that pyramidal cells exhibit inhibition-induced spike-timing resonance, without exhibiting cycle-averaged firing rate resonance [22]. Spiking fingerprints, as the ones presented by the 2D color images in **Fig 1CD**, are useful tools to visualize the possible occurrence of firing rate resonance. To generate a fingerprint, the number of spikes is counted at every relevant frequency and phase (over all trials), and divided by the time spent in that bin, yielding instantaneous rates.

Previously, spiking resonance generated in the noise-driven regime was quantified by computing the modulation of the instantaneous firing rate averaged over many trials in response to sinusoidal input (e.g., [11,19]). In the lack of noise, the modulation metric is insensitive to the number of spikes in every cycle. In the presence of high noise, the metric loses sensitivity to the precise phase. In contrast, the coherence metric is sensitive to both the number of spikes and the spike phase, both in the presence and in the lack of noise.

Both cycle-averaged firing rate resonance and spike timing resonance pertain to maximizing the output of the system at a non-zero input frequency. This is distinct from stochastic resonance [32,33], where the input-output relations are maximized at a non-zero level of noise (in the presence of an external input); and from coherence resonance [34–36], where the system exhibits maximally-coherent oscillations at a non-zero level of noise (in the absence of a periodic input).

In summary, resonance in the spiking domain can be visualized using fingerprinting and quantified using cycle-averaged firing rate, coherence, or both. From the perspective of a postsynaptic neuron, cycle-averaged firing rate resonance and spike timing resonance capture the input for neurons sensitive to firing rate and spike timing, respectively. When all (or at least most) spikes are generated directly by the input, the two types of spiking domain resonance coincide. This can be achieved in modeling studies and in controlled in vitro experiments in a relatively straightforward manner. However, when there are additional spurious spikes not created by the input as typically observed in vivo, resonance may appear and be detected only as spike timing resonance.

### Building blocks necessary for generating network resonance in neuronal systems

With the metrics for cycle-averaged firing rate and spike timing resonance in hand, we examine how resonance at one level of organization is related to frequency-dependent mechanisms at another level of organization. From an electrical circuit perspective, at least two building blocks are required for resonance to occur: (i) high-pass filtering, and (ii) low-pass filtering. Amplification within the band-pass may further enhance resonance. The building blocks and their interactions may be highly nonlinear. In neuronal systems, building blocks are realized by biophysical constructs which can have the same or distinct origins (e.g., distinct combinations of currents). The building blocks producing a given resonance may occur at the same or at distinct levels of organization (e.g., synaptic and spiking). In general, the frequency-dependent building blocks remain to be identified, and their interaction within and across levels of organization remains to be understood.

### Resonance generated at the subthreshold level can be inherited to the network level

**Fig 2:**
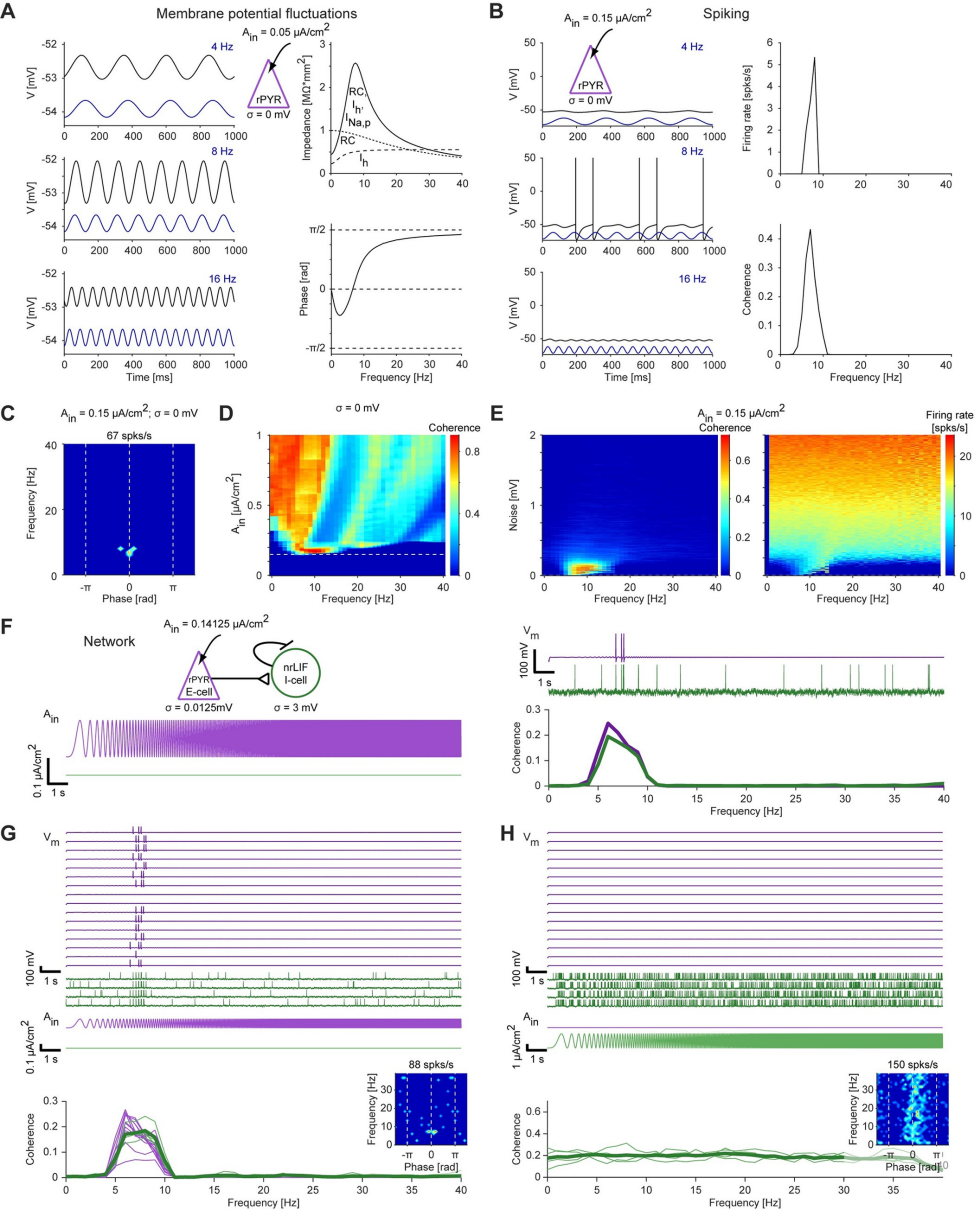
Resonance generated at the level of membrane potential fluctuations can be inherited to the network level. (**A**) A model neuron, consisting of leak current, persistent sodium current (*I*_*Na*,*p*_), h-current (*I*_*h*_), and threshold-based spiking with voltage reset, was driven by periodic current at various frequencies. Here and in **B-D**, *σ = 0 mV*. *Left*: Current input (dark blue traces, arbitrarily scaled) and membrane potential output (black traces) at three selected frequencies. *Top right*: Impedance profiles. A simplified model neuron with leak current and membrane capacitance shows only a low-pass filter (LPF) response (“RC”; dotted line). A simplified model with reduced capacitance shows only a high-pass filter (HPF) response (“*I*_*h*_”; dashed line). The full model shows resonance around 7–8 Hz (“*RC*, *I*_*h*_, *I*_*Na*,*p*_”). *Bottom right*: Phase of the membrane potential fluctuations at every frequency of the input current. (**B**) The model neuron of panel **A** was driven by higher-amplitude sinusoidal currents. *Left*: Spikes are produced specifically at the input frequency that corresponds to the peak of the impedance profile (panel **A**, top left). *Right*: The *I*_*Na*,*p*_*+I*_*h*_ spiking model neuron shows firing rate (*top*) and spike timing (*bottom*) resonance. (**C**) Spiking fingerprint (firing rate as a function of frequency and phase) for the same data as in panel **B**. Spikes occur at a specific frequency and near zero phase, corresponding to the co-occurrence of both cycle-averaged firing rate and spike timing resonance. (**D**) The model neuron was driven by input currents of various amplitudes (*A*_*in*_) while holding noise at zero (*σ = 0 mV*). Horizontal dashed line indicates the A_in_ value used in panels **B** and **C**. At higher *A*_*in*_ values the coherence becomes multi-modal. (**E**) The model neuron was driven by a fixed-amplitude input current (*A*_*in*_ *= 0*.*15 μA/cm*^*2*^) while varying membrane potential variability σ. Coherence (*left*) and firing rate (*right*) are shown as a function of noise magnitude. At higher noise magnitudes, spikes occur at all frequencies and spiking resonance is lost. (**F**) *Top left*: An E-cell, modeled by a *I*_*Na*,*p*_*+I*_*h*_ spiking neuron as in panel **A**, was connected via an excitatory (AMPA-like) synapse to a target I-cell, modeled as a non-resonant leaky integrate and fire (nrLIF) neuron. *Bottom left*: Constant-amplitude periodic current in the form of a linear chirp (0–40 Hz, 20 s) was applied to the E-cell (purple trace), that also received low-magnitude noise (*σ = 0*.*0125 mV*). The target cell received higher noise (*σ = 3 mV*). *Top right*: The target cell exhibits both background and transmitted spikes. *Bottom right*: Spiking resonance is observed for both model neurons. (**G**) *Top*: Voltage traces of four target I-cells (nrLIF; green) that received feedforward connections from 16 E-cells (*I*_*Na*,*p*_*+I*_*h*_ spiking; purple). All E-cells received exactly the same periodic input current; each cell received independent noise. *Bottom*: Coherence for every individual model cell (light traces), and averaged coherence for the target cells (heavy green trace). Spiking resonance is exhibited for the indirectly-activated target cells. *Inset*: spiking fingerprint for an I-cell. (**H**) The periodic input current was applied only to the I-cells; current amplitude was increased 16-fold; same network as in panel **G**. No spiking resonance is generated in the I-cells.

We begin with the best studied type of neuronal resonance, of membrane potential fluctuations (**Fig 2A**; sometimes referred to as “subthreshold” resonance; [1,2,5,6]). To determine whether subthreshold resonance can be inherited to the network level via spiking resonance, we first examine the communication of subthreshold level to the spiking level; and then study the communication from the spiking level to the network level. We modeled membrane potential resonance using a conductance-based neuron with leak, persistent sodium, and h-currents, augmented with threshold spiking and reset. In the *I*_*Na*,*p*_*+I*_*h*_ model, the subthreshold impedance profile peaked at 7.5 Hz (**Fig 2A, top right**). In this case, the LPF corresponds to the membrane capacitance and leak current (“RC”); the HPF, to the regenerative (h-) and leak currents; and the persistent sodium current acts primarily to amplify the band-pass response.

To understand whether and under what conditions resonance at the level of membrane potential fluctuations can be inherited to the network level, we increased the amplitude of the current input to the *I*_*Na*,*p*_*+I*_*h*_ model neuron. At the minimal input amplitude required to generate spikes (0.15 μA/cm^2^), the spikes occurred specifically around 7–8 Hz (**Fig 2B, left**), the same frequency at which the impedance profile peaked (**Fig 2A**). Spikes occurred near the zero phase of the input, so both cycle-averaged firing rate resonance and spike timing resonance were observed (**Fig 2B, right**; fingerprint at **Fig 2C**). To understand the conditions under which resonance is inherited to the spiking domain in the *I*_*Na*,*p*_*+I*_*h*_ model, we first modified input amplitude. We found that at higher amplitudes, spikes occurred coherently not only around 8 Hz but also at multiple other frequencies (**Fig 2D**). Second, we modified the amount of background inputs (noise; modeled by membrane potential variability, σ) in the model, while holding the input amplitude fixed at 0.15 μA/cm^2^. We used a range of noise levels between 0–2 mV, which is higher than observed during intracellular recordings using sharp electrodes from freely-moving mice [37]. Under high noise circumstances, spikes occurred at all frequencies and spiking resonance was lost (**Fig 2E**). Nevertheless, for a certain range of input amplitudes and noise levels, resonance at the level of membrane potential fluctuations is readily inherited to the spiking domain.

Next, we connected a resonant excitatory cell (E-cell; modeled as an *I*_*Na*,*p*_*+I*_*h*_ spiking neuron) via an excitatory (AMPA-like) synapse to a target cell, modeled as a leaky integrate and fire (LIF) neuron that did not exhibit subthreshold resonance (**Fig 2F**). The postsynaptic target LIF received relatively high background input (*σ = 3 mV*), and exhibited spontaneous spiking (**Fig 2F, top right**). When oscillatory chirp current was applied to the presynaptic neuron, the E-cell spikes induced additional spikes in the target cell, which displayed spiking resonance at the same frequency range as the presynaptic E-cell (**Fig 2F, bottom right**). We denote this phenomenon as “inherited network resonance”: resonance observed at the network level, which is inherited from frequency-dependent mechanisms at another level of organization. A similar pattern was observed in a larger network, consisting of 16 resonant E-cells that made feedforward excitatory connections on four non-resonant target cells (**Fig 2G**). Notably, in the same network, applying the oscillatory current directly to the target cells did not induce resonance in the target cells, even when current amplitude was increased (**Fig 2H**). In summary, resonance generated at the level of membrane potential fluctuations (**Fig 2A**) can be inherited to the spiking domain at low and intermediate noise levels (**Fig 2B–2E**). This extends previous modeling results linking subthreshold and spiking resonance [5,11] by showing that when input is very strong (**Fig 2D**) or when noise is very high (**Fig 2E**), subthreshold resonance is no longer communicated to the spiking level. Furthermore, subthreshold resonance can be inherited, via spiking resonance, to the network level (**Fig 2F–2G**).

### Resonance can be generated directly at the spiking level

**Fig 3:**
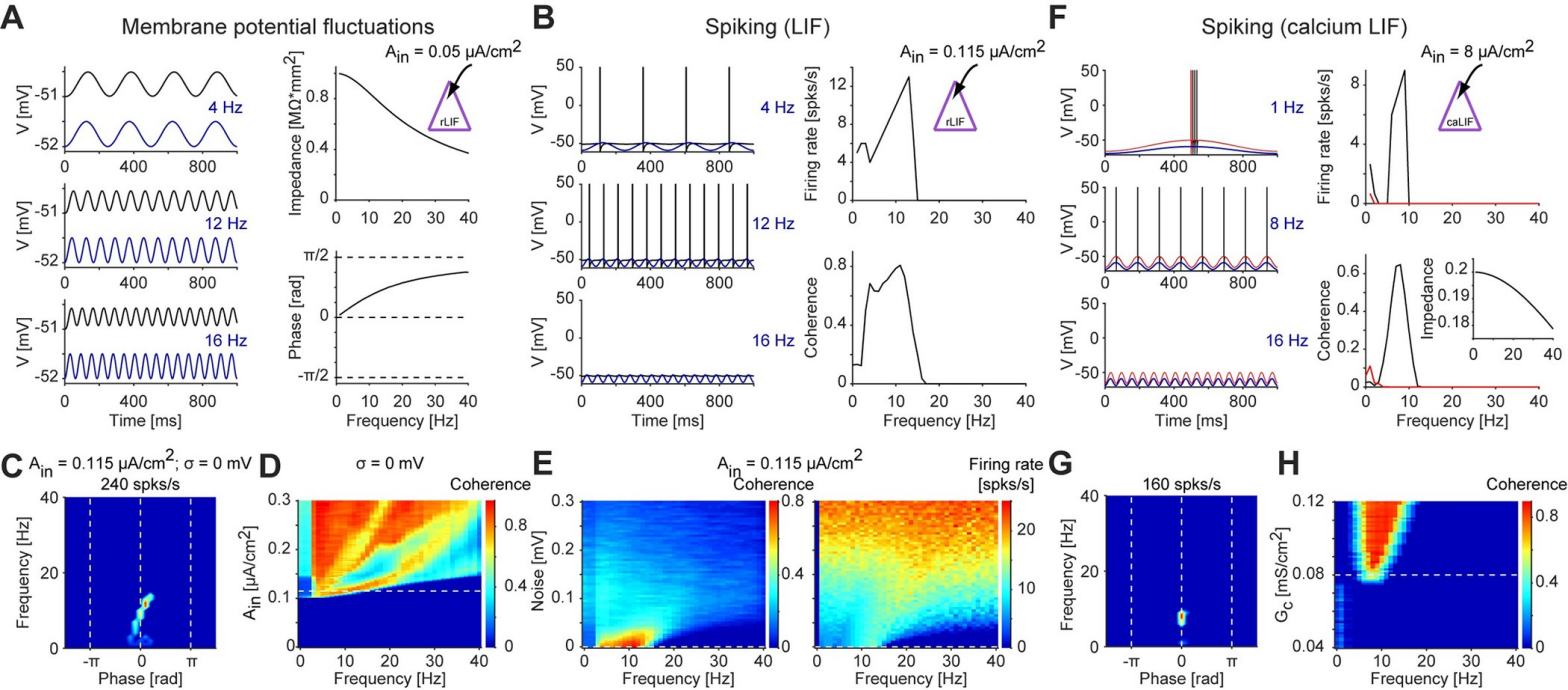
Resonance can be generated directly at the spiking level. (**A**) A leaky integrate and fire (LIF) model neuron was driven by periodic current at various frequencies. *Left*: Current input (blue, arbitrarily scaled) and membrane potential (black) at three selected frequencies. *Top right*: Impedance profile shows an LPF response. (**B**) The model neuron of panel **A** was driven by higher-amplitude periodic currents. *Left*: Spikes are produced at the peaks of the input cycles. At higher frequencies (e.g., 12 Hz), more cycles occur per unit time than at lower frequencies (4 Hz), corresponding to an HPF (discretization effect). *Right*: Combined with the subthreshold LPF (panel **A**), the “resonant LIF” (rLIF) exhibits spiking resonance. (**C**) Spiking fingerprint of the rLIF model; conventions are the same as in **Fig 2C**. Spikes are generated at a specific range of frequencies and phases, corresponding to spiking resonance. (**D**) Coherence as a function of input amplitude for the rLIF model; conventions are the same as in **Fig 2D**. At higher amplitudes, spikes occur at all input frequencies and the narrow-band resonance disappears. (**E**) Coherence (*left*) and firing rate (*right*) as a function of noise level, holding input amplitude fixed (*A*_*in*_ *= 0*.*115 μA/cm*^*2*^) for the rLIF model. When membrane potential variability increases, spikes occur at all input frequencies and the narrow-band resonance disappears. (**F**) A modified LIF neuron was constructed with spike dependent calcium dynamics (“calcium LIF”). The calcium-LIF model neuron has an LPF impedance profile (*bottom right*, *inset*). However, when driven by periodic current sufficient to generate spikes, the spikes appear at a specific frequency band (around 8 Hz; black traces). Without the calcium conductance, only a low-pass spiking filter remains (red traces). (**G**) Spiking fingerprint of the calcium-LIF model; conventions are the same as in **Fig 2C**. (**H**) Sensitivity analysis of the calcium-LIF to the calcium conductance G_c_. The width of the resonant frequency band increases with G_c_.

Conceptually, a subthreshold LPF generated by the passive (RC) properties of the membrane could interact with a spiking-domain HPF to generate spiking domain resonance. We therefore examined the HPF mechanism that underlies the generation of spiking resonance in the lack of resonance at the level of membrane potential fluctuations. First, we applied low-current input (0.05 μA/cm^2^) to a LIF model neuron without noise, which yielded an impedance profile corresponding to an LPF (**Fig 3A**). When current amplitude was increased (to 0.115 μA/cm^2^), spikes started to occur at the peaks of the oscillatory input cycles. Once a first spike is generated, the after-spike reset of the LIF prevents another spike from occurring until the membrane is recharged. If the cycle is sufficiently short, this results in only one spike per cycle, for a range of frequencies (**Fig 3B, left**). Since there are more cycles per unit time (e.g., second) at higher frequencies, the generation of a single spike per cycle automatically corresponds to high pass filtering. We identify the “spike discretization” effect as an HPF. Together with the subthreshold LPF (**Fig 3A**), the net outcome is spiking resonance (**Fig 3B, right; 3C**). Thus, consistent with earlier work [26–28], an isolated LIF model neuron can generate spiking resonance in the lack of noise. However, the band-pass (resonant) spiking response is generated by frequency-dependent mechanisms at two distinct levels of organization. Specifically, the subthreshold LPF interacts with a spiking HPF based on the discretization effect.

To determine the conditions under which spiking resonance can be generated in a LIF model neuron, we first modified the input current amplitude. We found that narrow-band resonance occurred only at a small range of input amplitudes (**Fig 3D**). Furthermore, when background noise was increased, spikes occurred at all input frequencies, and the narrow-band spiking resonance disappeared (**Fig 3E**; [26,28]). Thus, band-limited spiking resonance in an isolated LIF that lacks resonance of membrane potential fluctuations occurs only at a limited range of parameters.

The spiking resonance in the LIF model neuron involved a spiking-domain HPF based on the discretization effect, but spikes were consistently generated below the resonant frequency. Following a sodium spike, neurons exhibit a calcium transient: a rapid increase and slower decrease of calcium, which is the basis of calcium imaging [38]. We used the calcium transients to design a modified version of a LIF model neuron that includes spike-dependent calcium dynamics (**Fig 3F**). By construction, the calcium current activates only in the presence of spikes. Without the calcium current, the model exhibited only a LPF response in the subthreshold domain (**Fig 3F, bottom right inset**), and the spiking response exhibited a similar profile (**Fig 3F**, red lines). Adding the spike-dependent calcium dynamics did not change the subthreshold response, but a spiking band-pass filter emerged (**Fig 3F–3G**). During the calcium transient, the membrane potential was more depolarized, allowing the generation of a spike in response to a lower current input, effectively reducing spiking threshold. Thus, the occurrence of one spike favored the occurrence of another spike during a specific time window dictated mainly by the calcium activation and deactivation time constants. Thus, we identify the calcium transients as a second spiking-domain HPF. Combined with the subthreshold LPF, spiking resonance emerged (**Fig 3F–3G**). Increasing the calcium conductance widened the resonant band (**Fig 3H**). Together with spike discretization in the isolated LIF, the two case studies identify spiking HPFs. In particular, these cases demonstrate that spiking resonance can be generated directly at the spiking level, without resonance at the level of membrane potential fluctuations.

### Resonance generated directly at the spiking level can be inherited to the network level

**Fig 4:**
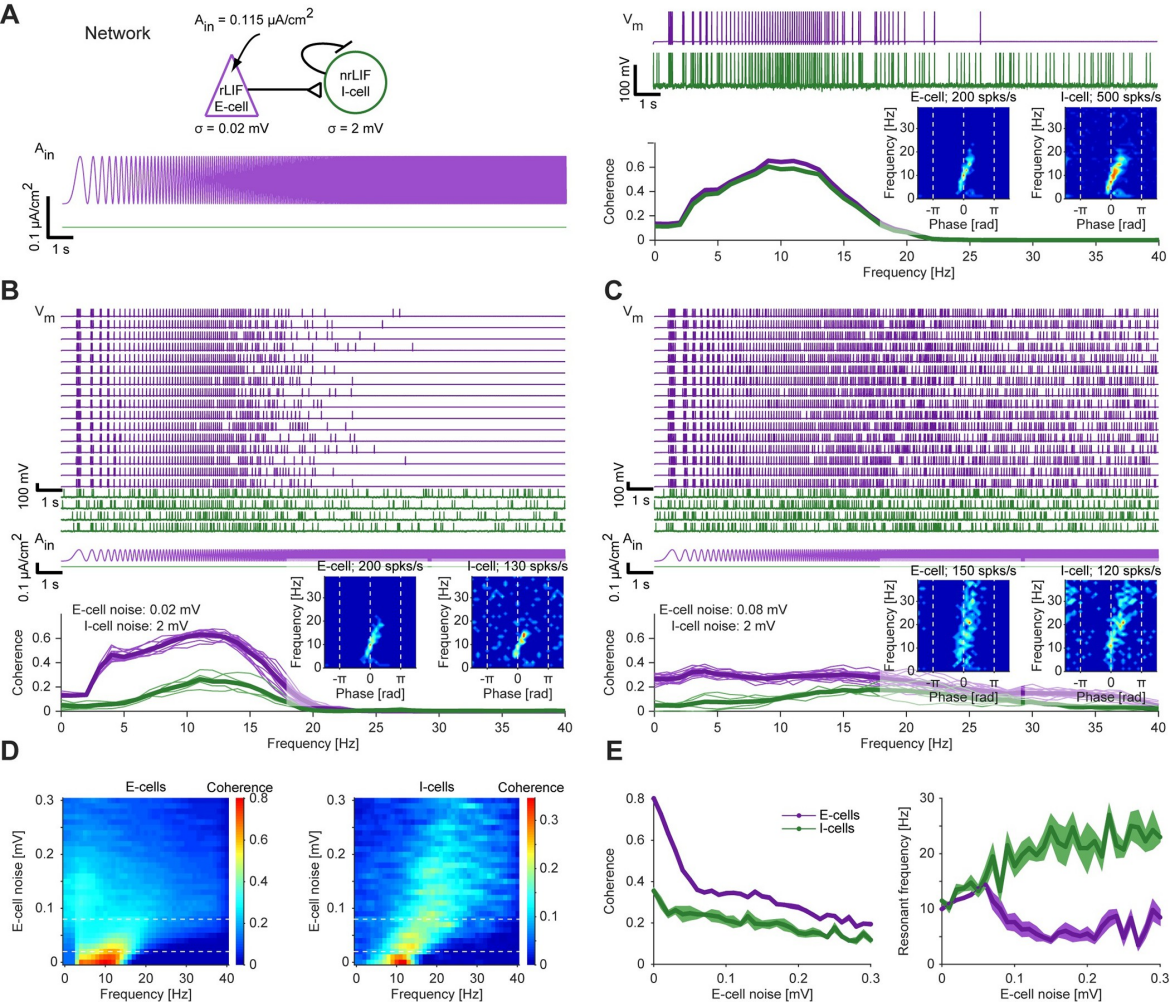
Resonance generated at the spiking level can be inherited to the network level. (**A**) *Top left*: An E-cell, modeled by an rLIF as in **Fig 3B**, was connected via an excitatory (AMPA-like) synapse to an I-cell, modeled by an nrLIF. *Bottom left*: Constant-amplitude periodic current in the form of a linear chirp was applied only to the E-cell (purple trace), that also received low-magnitude noise (*σ = 0*.*02 mV*). Here and in **B-E**, *A*_*in*_^*e*^ *= 0*.*115 μA/cm*^*2*^. *Top right*: The I-cell, that received higher magnitude noise (*σ = 2 mV*), exhibits both background and transmitted spikes. *Bottom right*: Spiking resonance is observed for both model neurons. *Inset*: Spiking fingerprints for an E-cell and for an I-cell. (**B**) *Top*: Voltage traces of four target I-cells (nrLIF; green) that received feedforward connections from 16 E-cells (rLIF; purple). All E-cells received exactly the same periodic input current; each cell received independent noise. *Bottom*: Coherence for every individual model cell (light traces), and averaged coherence for the E-cells (heavy purple traces) and the I-cells (heavy green traces). The indirectly-activated I-cells exhibit spiking resonance. *Inset*: Spiking fingerprints for an E-cell and for an I-cell. (**C**) The noise level to the E-cells was quadrupled (same network as in panel **B**). Spiking resonance of the I-cells is maintained, at a shifted (increased) resonant frequency. *Inset*: Spiking fingerprints for an E-cell and for an I-cell. (**D**) Coherence of the directly-activated E-cells (*left*) and the indirectly-activated I-cells (*right*), as the magnitude of the noise applied to the E-cell was varied systematically. Horizontal dashed lines indicate the E-cell noise levels used in panels **B** and **C**. Each row shows the average coherence (color coded) across 16 E-cells (*left*) or four I-cells (*right*). (**E**) Quantification of the maximal coherence magnitude (*left*) and the peak (“resonant”) frequency (*right*) for the dataset of panel **D**. Bands show SEM across cells. At low noise levels, E-cell and I-cell exhibit similar resonant frequencies.

To determine whether and how spiking resonance generated by a single LIF can propagate to other cells, we first connected the resonant LIF (“rLIF”; **Fig 3B**) as an E-cell to a postsynaptic target cell in a feedforward manner (**Fig 4A, top left**). The E-cell received a low level of membrane potential noise, keeping spiking within the resonant range (see **Fig 3E**). In contrast, the target cell was modeled as a non-resonant LIF (“nrLIF”) by increasing the membrane potential noise, and exhibited spontaneous spiking. When an oscillatory current input was applied to the E-cell, both the E-cell and the target cell displayed resonance (**Fig 4A, right**). The same phenomenon was observed in a larger network with feedforward excitatory connections: when current input was applied only to the E-cells, both the E-cells and the target cells exhibited resonance (**Fig 4B**; target cell fingerprint in **Fig 4B inset**). Thus, in a feedforward network of LIF neurons, network resonance emerges by inheritance from the spiking domain, without feedback or any additional frequency-dependent mechanisms at the synaptic or network levels. In previous work, spiking resonance was observed in recurrent LIF networks, in which E- and I-cells were connected with negative feedback [19]. The present observations show that network resonance can emerge in LIF networks without any recurrency or negative feedback, but rather by inheritance from resonance generated at the single neuron spiking level.

When the noise applied to the E-cells was quadrupled, coherence magnitude for both the E-cells and the target cells was reduced (**Fig 4C**), although spiking in the target cells was still limited to specific phases (**Fig 4C, inset**). With gradually increased noise, E-cell coherence gradually diminished (**Fig 4D–4E, left**), whereas the resonant frequency in the target cells gradually shifted to higher values (**Fig 4D–4E, right**). These results emphasize that even if resonance in a (LIF) network is entirely inherited from the single neuron spiking level, the properties of the single cell spiking resonance and network resonance may differ.

### Resonance generated at the synaptic level can be inherited to the network level

**Fig 5:**
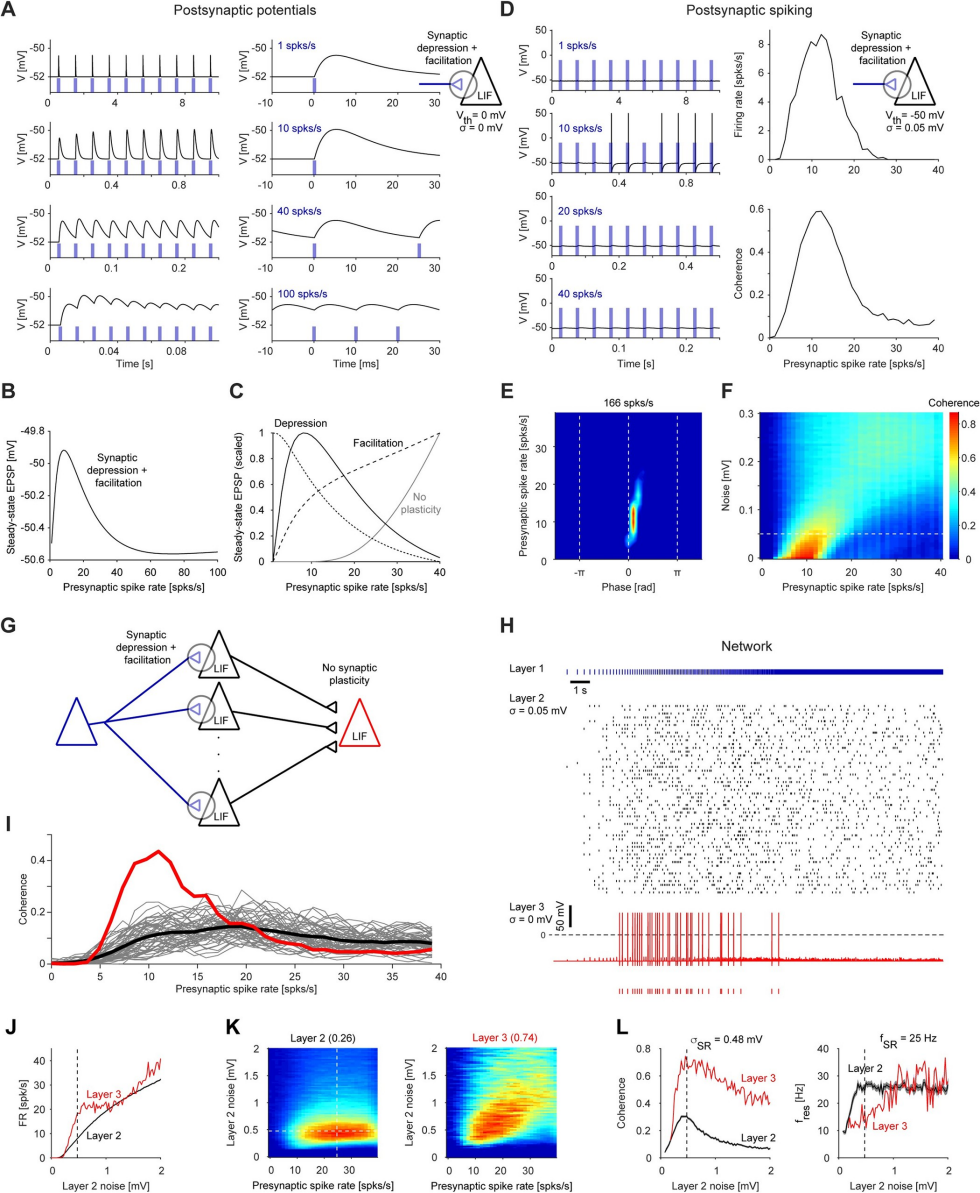
Resonance generated at the level of postsynaptic potentials can be inherited to the network level. (**A**) A LIF model neuron was driven by periodic spike trains at various rates via an excitatory (AMPA-like) synapse that exhibited synaptic depression and facilitation. Threshold was set to a high value (*V*_*th*_ *= 0 mV*) to prevent spiking. Here and in **B-C**, *σ = 0 mV*. *Left*: After several spikes, the excitatory postsynaptic potentials (EPSPs) stabilize. *Right*: Traces shown at an expanded time scale. The magnitude of the EPSPs is maximal at intermediate rates. (**B**) EPSP magnitude for the LIF with synaptic depression and facilitation, measured over a wide range of presynaptic spike rates. Magnitude peaks at an intermediate frequency, corresponding to synaptic resonance. (**C**) Scaled EPSP magnitude as a function of presynaptic spike rate for the LIF with synaptic depression and facilitation (black; same as in **B**). Scaled EPSP magnitudes for a synaptic plasticity model only with depression (dotted line) correspond to an LPF. Scaled EPSP magnitudes for a model only with facilitation (dashed line) or a model without synaptic plasticity (passive membrane; grey line) correspond to HPFs. (**D**) The LIF with synaptic resonance model neuron of panel **A** was modified to allow spiking (*V*_*th*_ *= -50 mV*). Here and in **E**, *σ = 0*.*05 mV; I*_*bias*_ *= 1*.*3 μA/cm*^*2*^. *Left*: Spikes are generated predominantly at intermediate frequencies. *Right*: The model exhibits spiking resonance. (**E**) Spiking fingerprint of the LIF with synaptic resonance model; conventions are the same as in **Fig 2C**. Spikes are generated at a specific range of frequencies and phases, corresponding to spiking resonance. (**F**) Coherence as a function of noise level. Dashed line indicates noise level of 0.05 mV, used in **D-E**. The resonant frequency (and coherence magnitude) shifts with increased noise. Spiking resonance is exhibited for a wide range of noise levels. (**G**) A diverging-converging feedforward network of LIF neurons was constructed. The first layer included a single point process neuron which fired a single spike at the peak of every cycle of a linear chirp (0–40 Hz over 20 s). The second layer included 50 identical LIF neurons with synaptic depression and facilitation (as in **D**); all neurons received excitatory (AMPA-like) connections from the layer 1 neuron, and every neuron received independent membrane potential noise. All layer 2 neurons received bias current of *I*_*bias*_ *= 1*.*2 μA/cm*^*2*^. The third layer included a single LIF without short term synaptic dynamics. (**H**) Neurons in the second layer spike at a wide range of input presynaptic spike rates, whereas the third layer (output) neuron spikes at a narrower range of presynaptic spike rates. (**I**) Second layer spike trains exhibit spiking resonance (thick black trace, averaged coherence over all inner-layer trains), consistent with noisy inheritance from the PSP level (as in **F**). The output spike train exhibits narrow-band network resonance (red trace). (**J**) The feedforward network was constructed and stimulated as in **G**, with different noise levels *(σ = 0–2 mV* at *0*.*025 mV* increments) received by layer 2 LIF neurons while keeping the noise received by the output (layer 3) neuron zero. The black curve shows the mean±SEM firing rate of the 50 layer 2 neurons. The vertical dashed line corresponds to the frequency for which layer 2 coherence peaks (**K**, *left*). (**K**) Peak coherence is observed for intermediate noise levels. Coherence between the input spike train (blue train in **H**) and the spike train of every layer 2 neuron was estimated and averaged over all 50 layer 2 neurons. The process was repeated for every noise level, and the coherence vectors are shown as rows in the left matrix (blue/red colors correspond to 0/0.26 coherence). The same process was carried out for the layer 3 neuron (right matrix; blue/red colors corresponding to 0/0.74 coherence). The white dashed lines correspond to the noise level and frequency for which layer 2 coherence peaks (0.3). (**L**) For every noise level, the peak layer 2 coherence magnitude (*left*) and the frequency for which the coherence peaks (*right*) are plotted. Layer 3 coherence magnitude is higher than layer 2 coherence for all noise levels. Layer 2 and layer 3 coherence peak at intermediate noise levels, exhibiting stochastic resonance. The resonant frequency of layer 3 is lower than the resonant frequency of layer 2 at every noise level, including at the stochastic resonant frequency (25 Hz for layer 2).

In addition to the level of membrane potential fluctuations (**Fig 2**) and the spiking level (**Fig 3**), resonance may be generated directly at the level of postsynaptic potentials (PSPs; [13–15,39]. Following the previous work, we modeled resonance at the PSP level using short-term synaptic dynamics (**Fig 5**). The model neuron was a LIF with a very high spiking threshold (leaky integrator), and input was given as periodic spike trains (without oscillatory current injection; **Fig 5A**). At the level of membrane potential fluctuations, the LIF exhibited only a low pass response (same as the LIF in **Fig 3A**). When short-term synaptic dynamics included both synaptic depression and facilitation, the excitatory PSP (EPSP) magnitude was highest around 8 Hz (**Fig 5A–5B**). This phenomenon is referred to as synaptic, or PSP, resonance [13–15]. In the depression/facilitation model of synaptic resonance, the LPF corresponds to synaptic depression (**Fig 5C, dotted line**) and the HPF corresponds to synaptic facilitation (**Fig 5C, dashed line**). Notably, when no synaptic plasticity was modeled, we identified an intrinsic synaptic HPF (**Fig 5C, grey**), consistent with temporal summation of multiple spikes by the membrane time constant. Thus, consistent with previous results [13,14], resonance at the level of postsynaptic potentials can be generated without resonance at the level of membrane potential fluctuations.

To determine whether PSP resonance can be inherited to the spiking level, we set the spiking threshold in the model LIF to a “standard” value (-50 mV). Under these conditions, the model neuron exhibited spiking resonance, at frequencies similar to those exhibited by the PSPs (**Fig 5D**). As for spiking resonance inherited from the subthreshold level (**Fig 2C**) and resonance generated directly at the spiking level (**Fig 3C, 3G**), the spiking resonance inherited from the PSP level occurred around zero phase (i.e., the input spikes; **Fig 5E**). In this case, a short phase lag occurred, consistent with synaptic delay (i.e., the rise time of the EPSP; **Fig 5A**). When the level of noise was increased, coherence magnitude was reduced, and the resonant frequency shifted to higher frequencies (**Fig 5F**). Thus, resonance generated at the level of postsynaptic potentials can be inherited to the spiking level.

Noisy LIF with synaptic resonance exhibit spiking resonance at a frequency higher than the PSP resonant frequency (**Fig 5F**). To examine the effect of PSP resonance on spiking resonance in a network of neurons, we constructed a diverging/converging feedforward network consisting of multiple noisy LIF with synaptic resonance that received the exact same input spike train (**Fig 5G**). Indeed, the cells exhibited spiking resonance at a frequency higher than the PSP resonant frequency (**Fig 5HI**). When these LIF converged on a common target, the target neuron exhibited resonance (**Fig 5HI**), at a frequency shifted back to the PSP resonant frequency. Thus, resonance generated at the level of postsynaptic potentials can be inherited to the network level.

In the model of network level synaptic resonance (**Fig 5G–5I**), the resonance of the output (layer 3) neuron is at a lower frequency and has lower coherence with the input, compared to the intermediate (layer 2) LIFs. To understand what the resonant peak of the layer 3 neuron depends on, we repeated the simulation while varying layer 2 noise levels (independent noise for every LIF). Increasing the noise of the layer 2 neurons (while keeping the noise of the output neuron zero) yielded monotonically increasing firing rates of both layers (**Fig 5J**). However, the coherence of both layers did not increase monotonically but rather peaked at an intermediate noise level (**Fig 5K**), exhibiting stochastic resonance [32,33,36]. Specifically, the maximal layer 2 coherence was obtained at a noise level of σ = 0.48 mV (σ = 0.25 mV was used in **Fig 5G–5I**). At that noise level, layer 2 coherence peaked (0.3) at a resonant frequency of 25 Hz, whereas layer 3 exhibited higher magnitude coherence (0.72) at a frequency of 17 Hz (**Fig R5L**). Thus, stochastic resonance, defined as an optimal response to an input at an intermediate noise level, can be observed in parallel to resonance, defined as a peak of the response at an intermediate frequency.

### Resonance can be generated intrinsically at the network level via excitatory inputs

**Fig 6:**
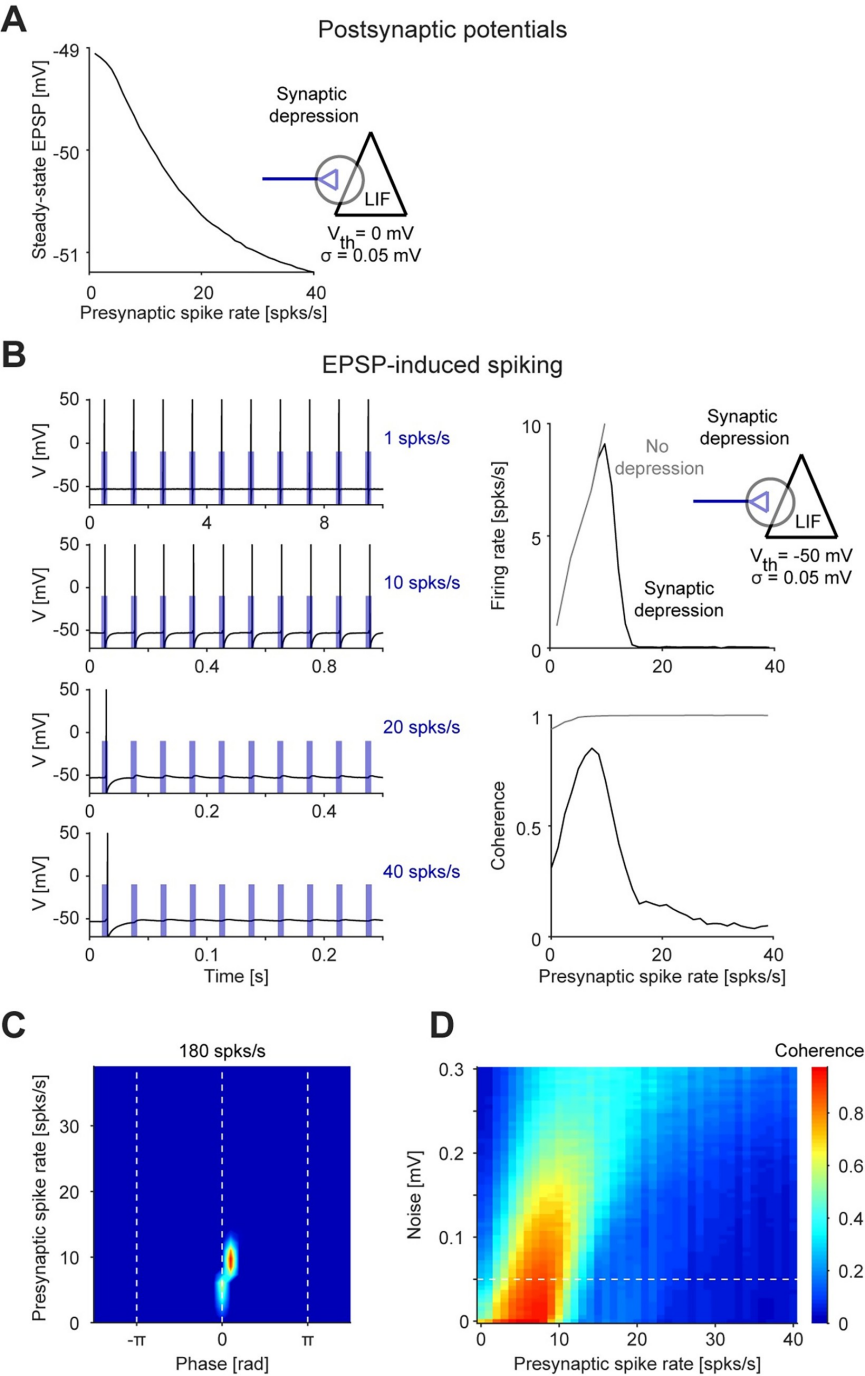
Intrinsic network resonance can be generated by combining frequency-dependent mechanisms at the level of postsynaptic potentials and at the spiking level. (**A**) EPSP magnitude for a LIF with synaptic depression (high threshold, *V*_*th*_ *= 0 mV*) as a function of presynaptic spike rate. Here and in **B-C**, *σ = 0*.*05 mV*. Without synaptic facilitation, EPSP magnitude is highest at the lowest rates, corresponding to a synaptic LPF. (**B**) The LIF with synaptic depression of panel **A** was modified to allow spiking (*V*_*th*_ *= -50 mV*). *Left*: Spike rate is highest at intermediate frequencies (e.g., 10 Hz). At higher frequencies (e.g., 20 Hz), spikes following the first spike are depressed. *Right*: In the LIF with synaptic depression model, the combination of the synaptic LPF (panel **A**) and the spike discretization HPF (grey line) yields spiking resonance (black line). Without synaptic depression, resonance disappears (grey line). (**C**) Spiking fingerprint of the LIF with synaptic depression model; conventions are the same as in **Fig 2C**. Spikes are generated at a specific range of frequencies and phases, corresponding to network resonance. (**D**) Coherence as a function of noise level. Dashed line indicates noise level of 0.05 mV, used in **B-C**. With increased noise, the resonant frequency shifts and coherence magnitude decreases. Spiking resonance is exhibited for a wide range of noise levels.

In principle, the frequency-dependent mechanisms (low- and high-pass filters) do not have to occur at the same level of organization. One example is spiking resonance in LIF, in which we identified the LPF as the membrane capacitance and leak current, and the HPF as spike discretization (**Fig 3B–3E**). To determine if frequency-dependent mechanisms across levels of organization can yield network resonance, we combined low-pass filtering at the PSP level and HPF at the spiking level. The PSP-level LPF was realized as synaptic depression (**Fig 6A**; cf. **Fig 5C, dotted line**). The HPF at the spiking level was manifested as spike discretization (grey curves in **Fig 6B, right**). When driven with presynaptic spike trains of various rates, the LIF with synaptic depression model exhibited spiking resonance (**Fig 6B, black lines**), with a resonant frequency around 7–8 Hz (**Fig 6B–6C**). Resonance was maintained in this model over a range of noise values, with a relatively small frequency shift (**Fig 6D**). We denote this phenomenon as “intrinsic network resonance”: resonance exhibited at the network level, in the lack of resonance observable at any other level of organization (around the frequency of interest). As in the previous three cases of network resonance (**Figs 2F–2H, 4,** and **5G–5I**), resonance is observed at the spiking level, in postsynaptic neurons. Yet in contrast to the cases of inherited network resonance, in the present case, no other level of organization exhibits resonance around the frequency of interest.

### Resonance inherited to the network level can be uncovered via inhibitory inputs

**Fig 7:**
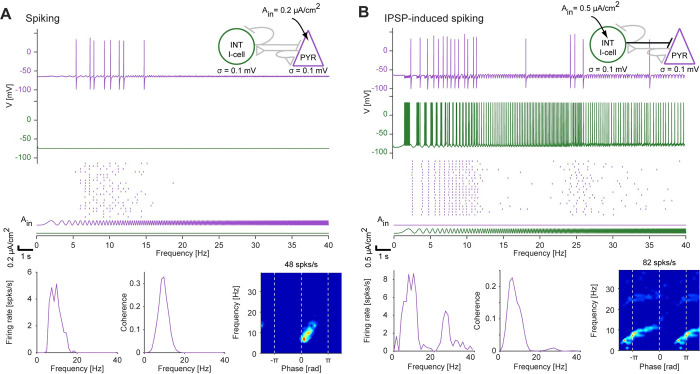
Inhibition-induced network resonance can be inherited from the level of membrane potential fluctuations. (**A**) A PYR model neuron, with h-current and full spiking dynamics, was driven by a constant-amplitude periodic current in the form of a linear chirp (0–40 Hz, 20 s; *A*_*in*_^*e*^ *= 0*.*2 μA/cm*^*2*^). *Top*: Membrane potential response during a single trial. *Center*: Raster plots from 20 independent trials. *Bottom*: Quantification of spiking resonance. As in the simpler model (**Fig 2**), the LPF and HPF correspond to RC (membrane capacitance and leak current) and the h-current, respectively. PYR spikes are generated around the peak of the input cycles in a narrow frequency band around 10 Hz, exhibiting spiking resonance. (**B**) The PYR model neuron of panel **A** was connected via an inhibitory (GABA_A_-like) synapse to a presynaptic I-cell (INT). Only the INT was driven by a constant amplitude periodic current (*A*_*in*_^*i*^ *= 0*.*5 μA/cm*^*2*^). Other possible synaptic connections were kept at zero (light grey lines in the cartoon, *top right*), isolating the contribution of feedforward inhibition. The PYR spikes after a series of INT spikes, around the trough of the input cycles given to the INT. The narrow-band PYR spiking exhibits IPSP-induced (network) resonance. All conventions are the same as in panel **A**.

Previous work showed that resonance can be observed in the spiking of postsynaptic neurons, i.e., at the network level, even when the synaptic connections are inhibitory [22]. When an isolated (subthreshold resonant) pyramidal cell (PYR), modeled with h-current and full spiking dynamics, was driven directly by a periodic input current, spiking resonance was generated (around 10 Hz; **Fig 7A**). This corresponds to resonance inherited from the level of membrane potential fluctuations, as observed in a simpler model neuron (**Fig 2**). We connected an I-cell, modeled with full spiking dynamics, to a resonant PYR (modeled as in **Fig 7A**) via an inhibitory (GABA_A_-like) synapse, without feedback. When only the I-cell in the two-cell model was driven, the PYR exhibited spiking resonance (around 8 Hz; **Fig 7B**). This network resonance is inherited from the PYR spiking resonance (**Fig 7A**), which was in turn inherited from resonance of the membrane potential fluctuations. Indeed, spike generation in the PYR required *I*_*h*_. However, the IPSP-induced PYR spikes occurred at the troughs of the input given to the I-cell (**Fig 7B, bottom right**), at an opposite phase compared to direct activation (**Fig 7A, bottom right**). This is consistent with in vivo observations [22] and contrasts with all other cases studied so far (membrane potential: **Fig 2C**; spiking: **Fig 3C**, **3G**; PSP: **Fig 5E**; EPSP network: **Fig 6C**), in which the resonant spikes occurred around the peak of the input cycle. Thus, network resonance can also be inherited from the single neuron level using synaptic inhibition.

**Fig 8:**
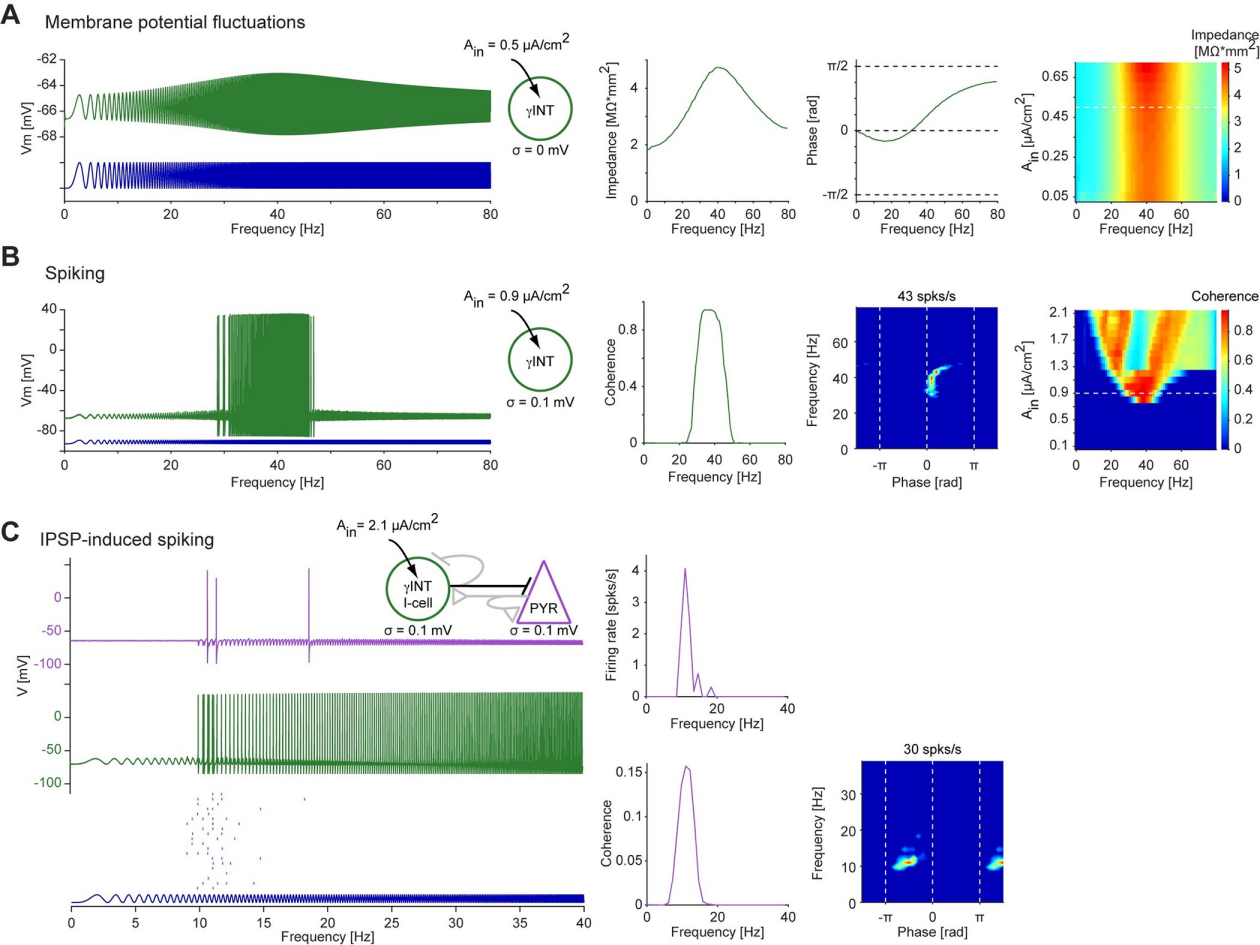
Inhibition-induced network resonance is sharpened by presynaptic high-pass filtering. (**A**) A gamma-interneuron (γINT) model neuron, with M-current and full spiking dynamics, was driven by constant amplitude periodic current in the form of a linear chirp (0–80 Hz, 10 s; *A*_*in*_^*i*^ *= 0*.*5 μA/cm*^*2*^). The impedance profile (second subpanel from left) shows a wide peak centered around 40 Hz, exhibiting resonance of the membrane potential fluctuations. (**B**) The γINT model neuron of panel **A** was driven by a higher-amplitude periodic current (0–80 Hz, 10 s; *A*_*in*_^*i*^ *= 0*.*9 μA/cm*^*2*^). Spikes are generated at the peaks of the input cycles, at a frequency band centered around 40 Hz (30–50 Hz). Thus, the γINT model neuron exhibits spiking resonance, inherited from the level of membrane potential fluctuations. *Far right*: Coherence as a function of input amplitude; horizontal dashed line indicates *A*_*in*_^*i*^ *= 0*.*9 μA/cm*^*2*^. At higher amplitudes, the spiking bandwidth increases. (**C**) The γINT model of panel **A** was connected, via an inhibitory (GABA_A_-like) synapse, to a PYR (as in **Fig 7B**), and driven by a constant amplitude linear chirp (0–40 Hz, 20 s; *A*_*in*_^*i*^ *= 2*.*1 μA/cm*^*2*^). *Top*: Membrane potentials during a single trial. As in **Fig 7B**, PYR spikes are generated after γINT spikes. However, the γINT spikes occur at higher input frequencies than the INT spikes, sharpening the PYR spiking resonance. *Center*: Raster plots of the PYR spikes from 20 independent trials. *Right*: Quantification of the IPSP-induced network resonance.

In the model of inhibition-induced network resonance (**Fig 7B**), the frequency-dependent mechanisms were inherited from the single-cell properties. Specifically, the PYR h-current acted as a HPF. Although the model exhibited resonance, spikes were also generated below and above the resonant frequency (**Fig 7B**). To construct a model of inhibition-induced network resonance that does not generate PYR spiking at low frequencies, we added a HPF at the level of the I-cell (**Fig 8**). This was done by modeling gamma-band resonance (previously observed in vitro; [8]) at the level of membrane potential fluctuations, by adding a resonant (M-) current to the I-cell. When driven with a periodic input current of low amplitude, the impedance profile of an isolated gamma-resonant interneuron (γINT) exhibited a peak (around 40 Hz; **Fig 8A**, right panels). When input amplitude was increased, the resonance generated at the level of membrane potential fluctuations was inherited to the spiking level. The peak coherence occurred at similar frequencies as resonance of membrane potential fluctuations (around 40 Hz), and the γINT spikes occurred around the input peak (zero phase; **Fig 8B**). Furthermore, when the γINT was connected to the PYR (modeled as in **Fig 7A**) via a single inhibitory synapse (as in **Fig 7B**), the PYR exhibited spiking resonance (around 10 Hz; **Fig 8C**). However, the phase of the PYR spikes (relative to the current input applied to the I-cell) differed in the two models of inhibition-induced network resonance (compare fingerprints in **Figs 7B** and **8C**). Furthermore, in the γINT network model, the produced PYR spikes were confined to the resonant frequency.

## Discussion

### Routes to network resonance

In this work, we tested the hypothesis that resonance in networks of spiking neurons is necessarily inherited from resonance at lower levels of organization. From electric circuit theory it is clear that one can construct a macro-circuit consisting of multiple embedded subcircuits, each being able to produce resonance on its own. However, neuronal networks are naturally evolved, highly nonlinear electric circuits which may not have an intrinsic resonance-generating property. This is primarily because the neuronal building blocks that determine the frequency-dependent properties (e.g., positive and negative feedback effects, history-dependent processes) rely on different biological substrates at different levels of organization (e.g., resonant and amplifying ionic currents, excitation and inhibition, synaptic depression and facilitation).

**Fig 9:**
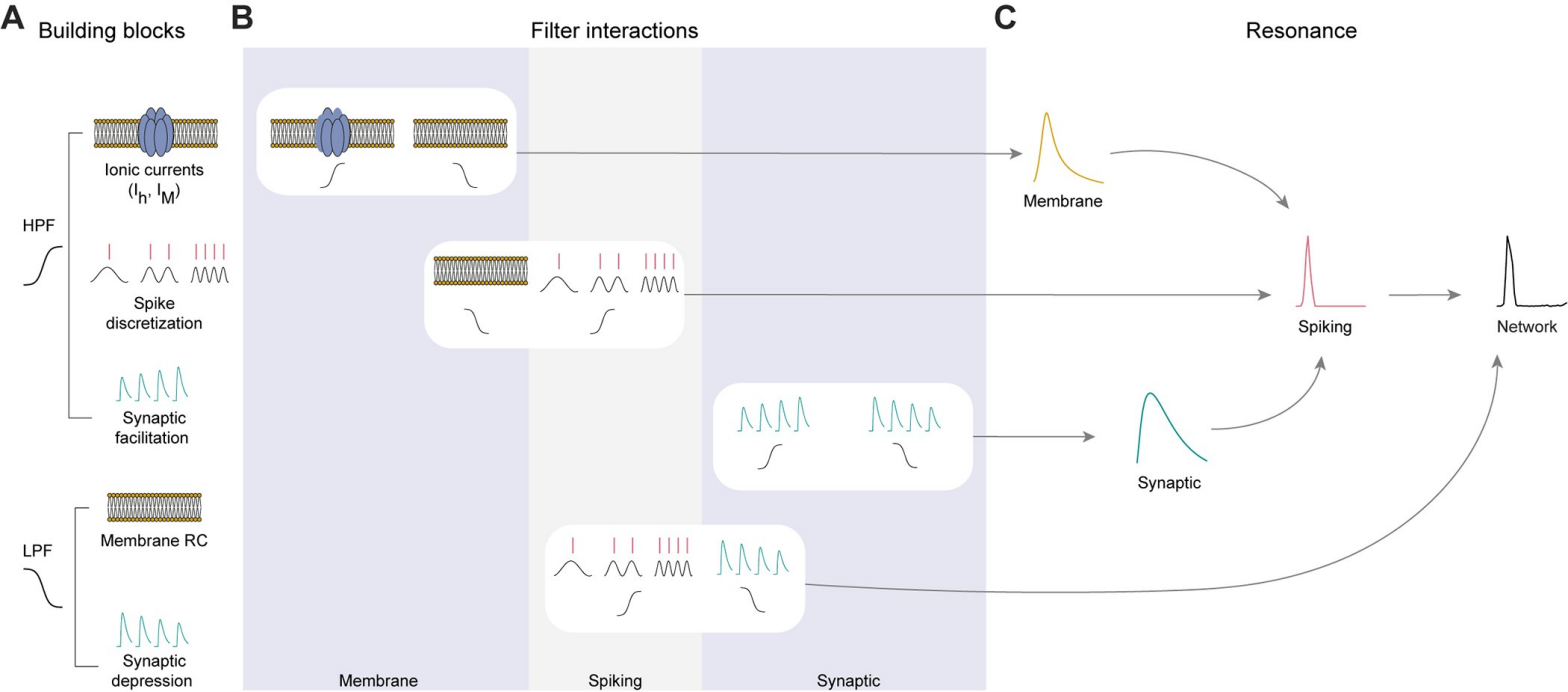
Network resonance can be generated by interacting low- and high-pass filters across levels of neuronal organization. (**A**) Frequency-dependent building blocks include high-pass filters (HPF, top) and low-pass filters (LPF, bottom). HPFs include inductive/resonant ionic currents (*I*_*h*_, **Figs 2, 7, 8**;, *I*_*M*_, **Fig 8**), acting at the level of membrane potential fluctuations; spike discretization and calcium-dependent spiking (**Figs 3, 4, 6**); and synaptic facilitation and temporal summation (**Fig 5**). LPFs include membrane capacitance and leak current (**Figs 2–4, 7, 8**), and synaptic depression (**Figs 5, 6**). (**B**) The frequency-dependent building blocks (filters) can interact either within the same level of organization (e.g., top row: membrane potential fluctuations; third row: postsynaptic potentials) or across levels of organization (e.g., second and fourth rows). (**C**) Interaction of HPF and LPF (within or across levels of organization) can generate resonance. If the interaction is within the same level of organization (e.g., membrane potential fluctuations), resonance can be generated at that level, and may (under certain conditions) be inherited to the network level (top pathway). Alternatively, network resonance may be generated intrinsically, by HPF and LPF across levels of organization (bottom pathway).

Examining four levels of neuronal organization and a number of representative case studies, we found that resonance can either be inherited from one level to another, or be generated independently at each and every level. In networks of spiking neurons, resonance can be generated directly at the network level. We showed that it is possible for a given system to display resonance at one level of organization–membrane potential fluctuations, postsynaptic potentials, single neuron spiking, or network–but not in others. Spiking resonance and resonance of postsynaptic potentials are not necessarily accompanied by resonance of membrane potential fluctuations, and network resonance can be generated without resonance at any other level of organization. Thus, the mechanisms that can generate neuronal resonance at different levels of organization are distinct (**Fig 9, center**). A direct implication of these observations is that when a system presents resonance at multiple levels of organization, these can be derived from either similar (inherited) or independent mechanisms. A second direct implication is that neuronal networks in different brain structures may exhibit qualitatively similar resonant properties by disparate mechanisms.

### General framework for nonlinear decomposition of resonance

Mechanistic studies aim to provide explanations of a given phenomenon in terms of a number of constituent building blocks whose choice depends on both the phenomenon and the desired level of explanation. For neuronal systems, there are a number of available sets of building blocks, but not all of them are appropriate for the investigation of resonance across levels of neuronal organization. The biophysical explanation, in terms of the ionic currents of the participating neurons, synaptic currents, short-term plasticity and other biological components, becomes extremely complex for larger networks. The same occurs for the dynamical systems explanation in terms of nonlinearities, time scales, and vector fields. Circuit building blocks such as positive and negative feedback loops are applicable to some, but not all levels of neuronal organization. For example, while subthreshold resonance results from negative feedback interactions between the membrane potential and restorative ionic currents, synaptic resonance results from history-dependent mechanisms.

Our results support the hypothesis that the set of LPFs and HPFs are appropriate building blocks to explain the generation of resonance (BPFs) and that this approach can be used irrespective of the level of organization, and across levels of organization. We further hypothesize that this approach is universal. In other words, to understand the generation of resonance at a given level of organization, one must identify the constituent LPFs and HPFs. From this perspective, the decomposition of BPFs into LPFs and HPFs is not a mere description of resonance, but rather an explanatory theoretical tool to understand resonance in terms of structural and functional building blocks. A deeper understanding might be achieved by linking filters with specific sets of building blocks (**Fig 9**). Provided that the technology exists, the filters may be identified experimentally by making the necessary perturbations. Therefore, understanding the generation of LPFs and HPFs in terms of the neuronal substrates contributes to the understanding of the biophysical and dynamic mechanisms underlying the generation of resonance.

The proposed LPF-HPF framework has the advantage of incorporating, within a single conceptual umbrella, disparate processes such as negative feedback processes (capacitive, leak, resonant, and amplifying currents), history-dependent processes (synaptic depression and facilitation), and spike discretization. It is not conceived as an analysis tool, but rather serves as a conceptual tool in which mechanistic models can be designed and their predictions tested by comparing modeling results to data. Further research is needed to explicitly integrate amplification in this framework, to establish a general LPF-HPF amplification framework for neuronal systems, and to identify the appropriate filters and amplification processes. Additional research is also needed to investigate the consequences of the interplay of multiple filters (e.g., two LPFs and one HPF) and across levels of organization, and to establish whether multiplicities produce degeneracies or richer patterns (e.g., anti-resonances).

The identification of the LPF and HPF constituting a given BPF is not a straightforward process, primarily due to two factors: the nonlinearities involved, which are typically strong; and the qualitatively different biophysical components operating at different levels of organization. In linear systems, for which analytical calculations are possible, the BPFs characterizing the presence of resonance can be generated by the frequency domain multiplication of LPFs and HPFs. These filters have been identified in simple neuronal systems (e.g., systems that can be described by RLC circuits), but it is not a-priori clear whether and how neuronal BPFs in general can be decomposed into LPFs and HPFs. Under rather general circumstances, for nonlinear subthreshold resonance one can extend the linear approach (in the time domain) and obtain a description of the LPF by disrupting the negative feedback from the recovery variable, and the HPF by neglecting the capacitive current. In contrast, the short-term plasticity-mediated synaptic BPFs that compose the synaptic resonance model are, by construction, the product of a depression LPF and a facilitation HPF in the time domain (not in the frequency domain), and are thus not amenable to linear decomposition.

In general, there are at least two possible ways to generate a resonant response at a given level of organization: by using an LPF and a HPF at the same level of organization, or at different levels (**Fig 9, center**). In the case of resonance of membrane potential fluctuations, we used a subthreshold LPF (passive membrane) and a subthreshold HPF (*I*_*h*_; **Fig 2**; [31]). Similarly, for synaptic resonance both the LPF (synaptic depression) and the HPF (facilitation) belonged to the same level of organization (**Fig 5**; [14]). However, for the generation of spiking resonance independently of resonance at any other level, we identified a mixed approach (**Fig 3**). While the HPF was spike-dependent (due to spike discretization or calcium dynamics), the LPF was inherited from the subthreshold domain (passive membrane). This provides a mechanistic explanation of the classical results of spiking resonance in LIF neurons [26,27], beyond the limit of weak inputs [28]. A mixed approach was also used for generating intrinsic network resonance (**Fig 6**): synaptic depression (LPF) was combined with spike discretization (HPF) to generate resonance in a postsynaptic target.

### Experimental and functional implications

Network resonance has been described theoretically [16,18–21]and observed experimentally [22,40,41] in several model systems. Here, we distinguished between two types of network resonance: “inherited” network resonance, and “intrinsic” network resonance. In inherited network resonance, frequency-dependent mechanisms (LPF and HPF) occur at a level of organization other than the network. Resonance can be observed at that level of organization, and may be inherited to the network level under specific conditions (e.g., **Fig 2**). Network-level processes may modulate (e.g., amplify or attenuate) the inherited resonance, but their absence does not disrupt the inherited resonance. In contrast, LPFs and HPFs that occur at possibly distinct non-network levels of organization can generate intrinsic network resonance (e.g., **Fig 6**), in the lack of resonance observable at any other level of organization. To the best of our knowledge, intrinsic network resonance has yet to be demonstrated experimentally.

Inhibition-induced network resonance required that I_h_-mediated rebound spiking in pyramidal cells [42] interacts with some form of HPF. Previously, depression of the inhibitory synapses (on the PYR) and interaction with a third type of cell (an oriens-lacunosum moleculare [OLM] cell) were suggested as HPFs [22]. Here, we considered two other mechanisms. First, we found that the PYR h-current itself yields a sufficient HPF for generating resonance in the IPSP-driven PYR. Thus, inhibition-induced network resonance can be inherited. Second, we found that the addition of a second HPF, in the form of gamma resonance in the presynaptic INT [43], sharpens the IPSP-induced PYR spiking resonance. Gamma resonance has been observed in computational models [16,21], in INT in vitro [8], and in multi-unit activity in vivo [41]. However, whether gamma resonance in INT actually occurs in vivo and sharpens theta-band resonance in PYR in vivo remains to be determined. Together, the present results suggest that although not necessary, frequency-modulating mechanisms at multiple levels of organization can contribute to the emergence of inhibition-induced network resonance.

Network resonance can be both intrinsic and inherited, and inherited network resonance can be derived from different levels of organization. By measuring only cycle-averaged firing rate resonance, it is impossible to determine the specific phase of the spiking response relative to a periodic input. However, using spike timing resonance and the fingerprint map of resonant neurons, different LPF and HPF modules that may underlie the resonance mechanism can be contrasted. One experimentally-testable prediction is that in recurrent excitatory networks, spiking resonance of directly-activated PYR will exhibit an earlier phase fingerprint, compared to the fingerprint of spikes generated via postsynaptic potentials which may be delayed in phase (**Figs 4B and 4C**, **6C**). Another experimentally-testable prediction is that in inhibition-induced resonance, PYR phase mediated by γINT would be later (**Fig 8C**), compared to PYR phase without the involvement of γINT (**Fig 7B**). Thus, in real neuronal networks driven by periodic inputs, spike timing resonance, quantified by spike phase and fingerprinting, may be used to dissect the frequency-dependent mechanisms underlying resonance.

Previous work suggested that resonance can optimize learning [44] and favor inter-neuronal communication [21]. We found that multiple routes can lead to network resonance. Thus, a single network could multiplex information from multiple sources. Multiplexing can occur at different resonant frequencies. Furthermore, since different types of network resonance exhibit different phases, multiplexing can also occur at different phases of the same frequency band.

### Related phenomena and future directions

We focused on resonance, defined as the maximal response of a system to periodic input in a limited frequency band, and left out the investigation of the related phenomenon of phasonance, defined as a zero-phase response to periodic inputs. Indeed, previous work has shown that frequency modulation of spike phase is possible using a LIF model with spike frequency adaptation provided by slower feedback, e.g., an outward calcium-activated potassium current [45]. Notably the calcium current used in the previous work (to show phasonance) provides subthreshold negative feedback, while the calcium current used in the calcium-LIF model (to show resonance; **Fig 3F–3H**) provides a suprathreshold positive feedback. For linear systems, phasonance (measured using the impedance phase) and resonance (measured using the impedance amplitude) can co-occur [11, 12]. However, phasonance does not have to accompany resonance (e.g., **Figs 5E** and **8C**), and when the two phenomena do co-occur, the resonant and phasonant frequencies do not necessarily coincide (they do for the case of the harmonic oscillator; [12]). As our results show, spiking resonance may be accompanied by spiking phasonance (**Fig 3BC**). In fact, spiking resonance and phasonance may be inherited from the subthreshold regime (**Fig 2BC**) or be generated at the spiking level (e.g., in LIF; **Fig 3BC**).

To address the main question of the paper we relied on a number of case studies. Further work is required to research general conditions under which resonance may be communicated from one level of organization to another, or generated independently at each level of organization. Future work should also consider the effects of multiple ionic currents in single neurons with possible heterogeneous spatial or compartmental distributions, the effects of interacting synaptic currents with different functions (excitation, inhibition), the effects of separate timescales and of short-term dynamic properties, and network topology effects. Additionally, future studies should consider scenarios in which multiple resonances interact within and across levels of organization.

### Conclusion

We have presented several novel computational models of representative scenarios, and have rejected the hypothesis that network resonance requires resonance at another level. While doing so, we set the infrastructure for a theoretical framework for investigating the mechanisms underlying the generation of neuronal network resonance, taking into account the interplay of the constitutive nonlinear properties of the participating neurons, synaptic connectivity, and network topology. This framework will enable studies of neuronal networks where the interactions between periodic inputs, currents, and network effects are important [46–48], different networks entrain each other [49,50], and/or the precise coordination between periodic input and spiking output are enhanced or disrupted [51–53].

## Materials and methods

### Models and numerical methods

We used biophysical (conductance-based) models, following the Hodgkin-Huxley formalism [54,55]. Models consisted of a set of coupled ordinary differential equations. A detailed description of the different models used is provided below. All numerical simulations were carried out using custom code written in MATLAB (The Mathworks, Natick, MA). Numerical integration was done using the explicit second-order Runge-Kutta endpoint (modified Euler) method [56] with integration time step *dt = 0*.*1 ms* (**Figs 1–6**) or *dt = 0*.*025 ms* (**Figs 7–8**) and simulation duration of *T s*. As current input, we used sinusoids of a single frequency, of the form

$$I_{in}(t)=I_{bias}+A_{in}\;\sin\left(2\pi ft\right)$$ (1)

or a chirp [6] linear in *f* of the form

$$I_{in}(t)=I_{bias}+A_{in}\;\cos\left(\pi +2\pi f_{0}t+\pi (f_{1}-f_{0})\frac{t^{2}}{T}\right)$$ (2)

Where *I*_*bias*_ is a time-independent (DC) bias current and *A*_*in*_ is the amplitude of the time-dependent (AC) periodic input. In the case of sinusoids of a single frequency *f*, input frequency *f* was typically varied from 1 Hz to 40 Hz at 1 Hz increments, and *T = 3 s*. For linear chirps, we typically used *f*_*0*_ *= 0 Hz* and *f*_*1*_ *= 40 Hz* with *T = 20 s*.

### Model for subthreshold resonance

To model resonance originating at the level of membrane potential fluctuations (**Fig 2A–2E**), we used a two-dimensional conductance-based model. Thus, the only ionic currents were persistent sodium with instantaneous activation (*I*_*Na*,*p*_), and h-current (*I*_*h*_) with voltage-dependent dynamics. In this model, low-pass filtering is induced by the membrane time constant (*C/g*_*L*_), high-pass filtering is induced by I_h_ and leak current, and amplification is provided by *I*_*Na*,*p*_. The model equations were:

$$C\frac{dV}{dt}=I_{in}(t)-g_{L}(V-E_{L})-g_{p}p_{\infty }(V)(V-E_{Na})-g_{h}r(V-E_{h})+g_{N}\eta (t)$$ (3)

$$\frac{dr}{dt}=\frac{r_{\infty }(V)-r}{\tau_{r}}$$ (4)

Membrane potential variability, which may stem from many unknown sources, was modeled by an additive white noise term, generated by random sampling from a zero-mean Gaussian distribution *η(t)~N(0*,*σ)*, multiplied by a constant conductance, *g*_*N*_ *= 1 mS/cm*^*2*^. The *I*_*h*_ time constant *τ*_*r*_ was assumed to be voltage-independent. The voltage-dependent activation/inactivation curves of the *I*_*h*_ and *I*_*Na,p*_ gating variables are given by:

$$p_{\infty }(V)=\frac{1}{1+e^{\frac{-(V+38)}{6.5}}}$$ (5)

$$r_{\infty }(V)=\frac{1}{1+e^{\frac{V+79.2}{9.78}}}$$ (6)

To model a passive membrane (**Fig 2A, dotted line**), we set the conductance of the persistent sodium (*g*_*p*_) and the h- (*g*_*h*_) currents to zero. To model a HPF (**Fig 2A, dashed line**), we set *g*_*p*_ to zero and reduced *C* to 0.1 μF/cm^2^. In all other cases, the full model was used.

Spike waveforms were not modeled explicitly, but a spike was said to occur whenever the membrane potential crossed a threshold value, *V*_*th*_. Thus, the 2D model was augmented with threshold spiking:

$$if\;V>V_{th}\;then\;V\leftarrow V_{reset}$$ (7)

Whenever a spike occurred, the membrane potential *V* was held constant at *V*_*peak*_ for *T*_*spike*_ before being reset to *V*_*reset*_. Following [12,57], the specific parameters values used were: *C = 1 μF/cm*^*2*^; *g*_*L*_ *= 0*.*1 mS/cm*^*2*^; *E*_*L*_ *= -65 mV*; *g*_*p*_ *= 0*.*1 mS/cm*^*2*^; *E*_*Na*_ *= 55 mV*; *g*_*h*_ *= 1 mS/cm*^*2*^; *E*_*h*_ *= -20 mV*; *τ*_*r*_ *= 100 ms*; *V*_*th*_ *= -50 mV*; *V*_*reset*_ *= -70 mV*; *V*_*peak*_ *= 50 mV*; *T*_*spike*_ *= 1 ms*; *σ = 0 mV* (**Fig 2E**: *σ = 0–2 mV*); *I*_*bias*_ *= -1*.*85 μA/cm*^*2*^; and *A*_*in*_ *= 0*.*15 μA/cm*^*2*^ (**Fig 2A**: *A*_*in*_ *= 0*.*05 μA/cm*^*2*^; **Fig 2D**: *A*_*in*_ *= 0–1 μA/cm*^*2*^).

### Model of an excitatory-inhibitory network

To model inheritance of resonance generated at the level of membrane potential fluctuations by *I*_*Na*,*p*_*+I*_*h*_ model neurons to postsynaptic targets (**Fig 2F–2G**), we generated a network of conductance-based E- and I-cells with all-to-all connectivity. All cells followed

$$C\frac{dV}{dt}=I_{in}(t)-g_{L}(V-E_{L})-I_{ionic}-I_{synaptic}+g_{N}\eta (t)$$ (8)

$$if\;V>V_{th}\;then\;V\leftarrow V_{reset}$$ (9)

The E-cells contained *I*_*Na*,*p*_ and *I*_*h*_, and thus ${I_{ionic}=g}_{p}p_{\infty }(V)(V-E_{Na})+g_{h}r(V-E_{h})$ with *r* obeying **Eq 4**. The I-cells were modeled as leaky integrate-and-fire (LIF) neurons, and thus *I*_*ionic*_ = 0. Synaptic connections were modeled as in [55,58]. For the e’th E-cell, the total synaptic current was

$$I_{synaptic,e}=\sum_{j=1}^{N_{e}}g_{ee}S_{ej}(V_{e}-E_{se})+\sum_{k=1}^{N_{i}}g_{ei}S_{ek}(V_{e}-E_{si})$$ (10)

Where *N*_*e*_ (*N*_*i*_) is the number of E-cells (I-cells). The notation *g*_*ej*_ indicates the maximal synaptic conductance from presynaptic E-cell *j* to postsynaptic E-cell *e*. All excitatory-to-excitatory synapses had the same maximal conductance values *g*_*ee*_ and reversal potentials *E*_*se*_, regardless of the presynaptic neuron. All inhibitory-to-excitatory synapses had the same maximal conductance values *g*_*ei*_ and reversal potentials *E*_*si*_, regardless of the presynaptic neuron. All synaptic activation variables corresponding to the same presynaptic neuron had the same dynamics, regardless of the postsynaptic neuron (*S*_*ej*_ *= S*_*j*_, *S*_*ek*_ *= S*_*k*_, *∀e*). For the i’th I-cell, the total synaptic current was modeled by

$$I_{synaptic,i}=\sum_{j=1}^{N_{e}}g_{ie}S_{ij}(V_{i}-E_{se})+\sum_{k=1}^{N_{i}}g_{ii}S_{ik}(V_{i}-E_{si})$$ (11)

All excitatory-to-inhibitory synapses had the same maximal conductance values *g*_*ie*_ and reversal potentials *E*_*se*_. All inhibitory-to-inhibitory synapses had the same maximal conductance values *g*_*ii*_ and reversal potentials *E*_*si*_. All synaptic activation variables corresponding to the same presynaptic neuron had the same dynamics (*S*_*ij*_ *= S*_*j*_, *S*_*ik*_ *= S*_*k*_, *∀i*).

For an excitatory/inhibitory presynaptic neuron, the dynamics of the corresponding synaptic variable (*S*_*e*_*/S*_*i*_) depended on the presynaptic membrane potential (*V*_*e*_*/V*_*i*_) and the synaptic rise and decay time constants, following:

$$\frac{dS_{e}}{dt}=H(V_{e})\frac{\left(1-S_{e}\right)}{\tau_{r}^{e}}-\frac{S_{e}}{\tau_{d}^{e}}$$ (12)

$$\frac{dS_{i}}{dt}=H(V_{i})\frac{\left(1-S_{i}\right)}{\tau_{r}^{i}}-\frac{S_{i}}{\tau_{d}^{i}}$$ (13)

H(V)=(1+tanh(V/4))/2 (14)

Parameter values followed [58]. All parameters values used are detailed in **Table 1**.

**Table 1:** Parameters used for modeling inheritance of resonance generated at the level of membrane potential fluctuations (Fig 2F–2H).

Parameter	Value	Units	Notes
C	1	μF/cm^2^	
g_L_	0.1	mS/cm^2^	
V_th_	-50	mV	
E_L_^e^	-65	mV	E-cells
g_p_	0.1	mS/cm^2^	E-cells
E_Na_	55	mV	E-cells
g_h_	1	mS/cm^2^	E-cells
E_h_	-20	mV	E-cells
τ_h_	100	ms	E-cells
V_reset_^e^	-70	mV	E-cells
T_spike_^e^	1	ms	E-cells
E_L_^i^	-60	mV	I-cells
V_reset_^i^	-60	mV	I-cells
T_spike_^i^	0.1	ms	I-cells
τ_r_^e^	0.1	ms	AMPA
τ_d_^e^	3	ms	AMPA
E_e_	0	mV	AMPA
τ_r_^i^	0.3	ms	GABA_A_
τ_d_^i^	9	ms	GABA_A_
E_i_	-80	mV	GABA_A_
g_ie_	0.05	mS/cm^2^	E to I; **Fig 2F**: 1
g_ee_	0	mS/cm^2^	E to E
g_ei_	0	mS/cm^2^	I to E
g_ii_	0.05	mS/cm^2^	I to I
σ^e^	0.0125	mV	E-cells
I_bias_^e^	-1.85	μA/cm^2^	E-cells
A_in_^e^	0.14125	μA/cm^2^	**Fig 2H**: 0
σ^i^	3	mV	I-cells
I_bias_^i^	-1	μA/cm^2^	I-cells
A_in_^i^	0	μA/cm^2^	**Fig 2H**: 2.26

### Models for spiking resonance

To model spiking resonance generated by an isolated LIF (**Fig 3A–3E**), we used

$$C\frac{dV}{dt}=I_{in}(t)-g_{L}(V-E_{L})+g_{N}\eta (t)$$ (15)

$$if\;V>V_{th}\;then\;V\leftarrow V_{reset}$$ (16)

with the following parameter values: *C = 1 μF/cm*^*2*^; *g*_*L*_ *= 0*.*1 mS/cm*^*2*^; *E*_*L*_ *= -60 mV*; *V*_*th*_ *= -50 mV*; *V*_*reset*_ *= -60 mV*; *V*_*peak*_ *= 50 mV*; *T*_*spike*_ *= 1 ms*; *σ = 0 mV* (**Fig 3E**: *σ = 0–0*.*3 mV*); *I*_*bias*_ *= 0*.*9 μA/cm*^*2*^; and *A*_*in*_ *= 0*.*05–0*.*3 μA/cm*^*2*^.

To model spiking resonance generated directly at the spiking level with a sharper HPF than the isolated LIF (**Eqs 15–16**), we modified the LIF model to include a spike-dependent calcium current (**Fig 3F–3H**). The model equations were:

$$C\frac{dV}{dt}=I_{in}(t)-g_{L}(V-E_{L})-g_{C}K(V-E_{Ca})+g_{N}\eta (t)$$ (17)

$$\frac{dK}{dt}=\frac{N_{C}(1-K)}{\tau_{act}}-\frac{K}{\tau_{inact}}$$ (18)

$$\frac{dN_{C}}{dt}=-\frac{N_{C}}{\tau_{deact}}$$ (19)

$$if\;V>V_{th}\;then\;\left\{ \begin{array}{c} V\leftarrow V_{reset}\\ N_{C}\leftarrow N_{reset} \end{array}\right.$$ (20)

The purpose of constructing this model was to generate a spike-dependent HPF, in a system that has an underlying subthreshold LPF. The physiological rationale is that following a spike, there is increased calcium influx, further increasing depolarization; this effectively reduces the spiking threshold to current input at the same level. Thus, at another cycle of input that occurs shortly after the first spike, there will be another spike–even if the current is insufficient to generate a spike without the calcium influx. However, if the next cycle occurs later, the intracellular calcium level will have already gone back to steady-state level.

In the model, the calcium gating variable *K* is limited to the [0,1] range and represents the probability of the gate to be open. Once a spike occurs, *N*_*C*_ is instantaneously reset to a non-zero value (*N*_*reset*_) and then slowly decays (with *τ*_*deact*_) towards zero. While *N*_*C*_ is non-zero, the gate opens slowly (i.e., *K* is activated towards 1 with *τ*_*act*_/*N*_*C*_, and rapidly inactivates (decays to zero with *τ*_*inact*_). When activation is very fast or inactivation is very slow, the calcium conductance remains high long after a spike, providing additional depolarization at multiple current input frequencies, generating spike bursts at every input cycle. When the activation is slow and inactivation is fast, *K* remains relatively high only for a short time after a spike. The parameters used favor the latter scenario. Specific parameter values were: *C = 1 μF/cm*^*2*^; *g*_*L*_ *= 0*.*5 mS/cm*^*2*^; *E*_*L*_ *= -60 mV*; *g*_*C*_ *= 0*.*08 mS/cm*^*2*^ (**Fig 3H**: *g*_*C*_ *= 0*.*04–0*.*12 mS/cm*^*2*^); *E*_*Ca*_ *= 100 mV*; *τ*_*act*_ *= 50 ms*; *τ*_*inact*_ *= 5 ms*; *τ*_*deact*_ *= 70 ms*; *V*_*th*_ *= -50 mV*; *V*_*reset*_ *= -70 mV*; *V*_*peak*_ *= 50 mV*; *N*_*reset*_ *= 0*.*1*; *σ = 0*.*001 mV*; *I*_*bias*_ *= -3 μA/cm*^*2*^; and *A*_*in*_ *= 8 μA/cm*^*2*^.

To model network resonance inherited from resonance generated at the spiking level (**Fig 4**), we combined a set of LIF model neurons (**Eq 15** and **Eq 16**) using the network formalism described above (**Eqs 8–14**), with parameter values as detailed in **Table 1**.

**Table 2:** Parameters used for modeling inheritance of spiking resonance generated by an isolated LIF (Fig 4).

Parameter	Value	Units	Notes
C	1	μF/cm^2^	
g_L_	0.1	mS/cm^2^	
E_L_	-60	mV	
V_th_	-50	mV	
V_reset_	-60	mV	
T_spike_	1	ms	
τ_r_^e^	0.1	ms	AMPA
τ_d_^e^	3	ms	AMPA
E_e_	0	mV	AMPA
τ_r_^i^	0.3	ms	GABA_A_
τ_d_^i^	9	ms	GABA_A_
E_i_	-80	mV	GABA_A_
g_ie_	0.01	mS/cm^2^	E to I; **Fig 4A**: 1
g_ee_	0	mS/cm^2^	E to E
g_ei_	0	mS/cm^2^	I to E
g_ii_	0.05	mS/cm^2^	I to I
σ^e^	0.02	mV	**Fig 4C**: 0.08 **Fig 4DE**: 0–0.3
I_bias_^e^	0.9	μA/cm^2^	E-cells
A_in_^e^	0.115	μA/cm^2^	E-cells
σ^i^	2	mV	I-cells
I_bias_^i^	0	μA/cm^2^	I-cells
A_in_^i^	0	μA/cm^2^	I-cells

### Models for synaptic plasticity and resonance

To model resonance generated at the level of postsynaptic potentials (**Fig 5**), we used a LIF model receiving a synaptic current with short term dynamics (synaptic facilitation and depression):

$$C\frac{dV}{dt}=I_{in}(t)-g_{L}(V-E_{L})-g_{S}SDF(V-E_{S})$$ (21)

$$\frac{dS}{dt}=H(V_{pre})\frac{\left(1-S\right)}{\tau_{r}}-\frac{S}{\tau_{d}}$$ (22)

$$\frac{dD}{dt}=-H(V_{pre})\frac{D}{\tau_{reset(d)}}+\frac{\left(1-D\right)}{\tau_{dep}}$$ (23)

$$\frac{dF}{dt}=H(V_{pre})\frac{\left(1-F\right)}{\tau_{reset(f)}}-\frac{F}{\tau_{fac}}$$ (24)

The threshold spiking is defined by **Eq 9** and the sigmoid activation function is as in **Eq 14**. In **Eqs 21–24**, *V*_*pre*_ represents the membrane potential of the presynaptic neurons. To construct the input *V*_*pre*_, we generated a spike at each local maximum of a sinusoid function (**Eq 1** or **Eq** 2). The presynaptic voltage was then defined as *V*_*pre*_*(t) = 50 mV* if a spike occurred in the last *1 ms*; otherwise, *V*_*pre*_*(t) = -60 mV*. Other specific parameter values used in **Fig 5** were: *C = 1 μF/cm*^*2*^; *g*_*L*_ *= 0*.*1 mS/cm*^*2*^; *E*_*L*_ *= -65 mV*; *V*_*th*_ *= -50 mV* (**Fig 5A**: *V*_*th*_ *= 0 mV*); *V*_*reset*_ *= -70 mV*; *T*_*spike*_ *= 0*.*1 ms*; *τ*_*r*_ *= 0*.*1 ms*; *τ*_*d*_ *= 3 ms*; *g*_*S*_ *= 0*.*175 mS/cm*^*2*^; *E*_*S*_ *= 0 mV*; *τ*_*reset(d)*_ *= 0*.*1 ms*; *τ*_*dep*_ *= 100 ms*; *τ*_*reset(f)*_ *= 0*.*2 ms*; *τ*_*fac*_ *= 300 ms*; *σ = 0*.*05 mV* (**Fig 5A–5C**: *σ = 0 mV*; **Fig 5F**: *σ = 0–0*.*3 mV*); *I*_*bias*_ *= 1*.*3 μA/cm*^*2*^; and *A*_*in*_ *= 0 μA/cm*^*2*^.

To model synaptic depression, the synaptic variable *S* was multiplied by a factor *D*, limited to the [0,1] range. After every spike, *D* slowly recovers towards its steady state value of 1, with time constant *τ*_*dep*_, which determines the time scale of depression (**Eq 23**). Since additional spikes may occur during recovery, the process is history-dependent. To model synaptic facilitation, the synaptic variable *S* was multiplied by a factor *F*, also limited to the [0,1] range. The dynamics of *F* follow the same principle as for depression (**Eq 24**), yet in an opposite direction: during every spike, *F* rapidly increases towards 1; between spikes, *F* relaxes to zero with a slower time constant *τ*_*fac*_. Note that in principle, the synaptic variable *S* in **Eq 22** is also history-dependent, representing synaptic summation. However, the synaptic decay time constant *τ*_*d*_ for the AMPA-like synapses used in **Eq 22** is much smaller than the time constants used for modeling depression.

To model the combined effect of depression and facilitation, the synaptic variable was multiplied by *D* and *F*. Together, the product *DF* represents the probability of presynaptic release. We note that the depression model is similar to the one proposed by [59]. Previous models of synaptic plasticity ([13, 60], attributed to Dayan, Abbott, and collaborators) included a discrete (delta-function) rise of the depression and facilitation variables in response to each presynaptic spike. The present synaptic plasticity models replace the step increase with a continuous sigmoid function, as previously used for synaptic transmission models [55, 58].

To model short term synaptic dynamics in the lack of depression/facilitation (**Fig 5C**), we set the corresponding variable to a constant (only facilitation: *D = 1*; only depression: *F = 1*).

To model inheritance of resonance generated at the level of postsynaptic potentials to postsynaptic targets (**Fig 5G–5L**), we constructed a 3-layer diverging/converging feedforward network. Synaptic conductance between layer 1 and layer 2 was *g*_*S*_ *= 0*.*2 mS/cm*^*2*^. Neurons in the second layer received *I*_*bias*_ *= 1*.*2 μA/cm*^*2*^ and independent noise (*σ* = 0.25 *mV* in **Fig 5G–5I**). Synaptic conductance between layer 2 and layer 3 was *g*_*S*_ *= 0*.*12 mS/cm*^*2*^; the single layer 3 neuron received *I*_*bias*_ *= 0 μA/cm*^*2*^ and no additional noise.

To model EPSP-induced network resonance (**Fig 6**), we used the LIF model supplemented with synaptic plasticity (**Eqs 9**, **14**, **21–24**), without facilitation (i.e., *F = 1*). Other parameter values were the same as for generating resonance at the level of PSP (**Fig 5**), with *I*_*bias*_ *= 1*.*2 μA/cm*^*2*^.

### Models for inhibition-induced network resonance

To model IPSP-induced network resonance (**Figs 7–8**), we used a minimal network of conductance-based neurons of the Hodgkin-Huxley type with instantaneous activation of sodium channels, consisting of an excitatory cell (a PYR) and an INT [58]. The PYR model included dynamics on the membrane potential (*V*^*e*^), sodium inactivation (*h*), delayed-rectifier potassium (*n*), and the h-current gating variable (*r*; [9,61]), yielding a 4D system. In addition, the model included synaptic input and noise. Denoting the membrane potential of the PYR by *V*^*e*^ and the membrane potential of the INT by *V*^*i*^, the full model for the PYR reads

$$C\frac{dV^{e}}{dt}=I_{in}^{e}(t)-g_{L}^{e}(V^{e}-E_{L}^{e})-g_{Na}^{e}h{m_{\infty }(V^{e})}^{3}(V^{e}-E_{Na}^{e})-g_{K}^{e}n^{4}(V^{e}-E_{K}^{e})-g_{h}^{e}r(V^{e}-E_{h}^{e})-g_{ee}S_{e}(V^{e})(V^{e}-E_{e})-g_{ei}S_{i}(V^{i})(V^{e}-E_{i})+g_{N}\eta^{e}(t)$$ (25)

$$\frac{dh}{dt}=\frac{h_{\infty }(V^{e})-h}{\tau_{h}(V^{e})}$$ (26)

$$\frac{dn}{dt}=\frac{n_{\infty }(V^{e})-n}{\tau_{n}(V^{e})}$$ (27)

$$\frac{dr}{dt}=\frac{r_{\infty }(V^{e})-r}{\tau_{r}(V^{e})}$$ (28)

The gating variables (*x = h*,*m*,*n*,*r*) had voltage-dependent time constants (*τ*_*x*_) and steady-state values (*x*_*∞*_) as follows:

$$h_{\infty }(V)=\frac{{0.128e}^{\frac{-(V+50)}{18}}}{{0.128e}^{\frac{-(V+50)}{18}}+\frac{4}{1+e^{\frac{-(V+27)}{5}}}},\tau_{h}(V)=\frac{1}{{0.128e}^{\frac{-(V+50)}{18}}+\frac{4}{1+e^{\frac{-(V+27)}{5}}}}$$ (29)

$$m_{\infty }(V)=\frac{\frac{0.32(V+54)}{1-e^{\frac{-(V+54)}{4}}}}{\frac{0.32(V+54)}{1-e^{\frac{-(V+54)}{4}}}-\frac{0.28(V+27)}{1-e^{\frac{\left(V+27\right)}{5}}}}$$ (30)

$$n_{\infty }(V)=\frac{\frac{0.032(V+52)}{1-e^{\frac{-(V+52)}{5}}}}{\frac{0.032(V+52)}{1-e^{\frac{-(V+52)}{5}}}+{0.5e}^{\frac{-(V+57)}{40}}},\tau_{n}(V)=\frac{1}{\frac{0.032(V+52)}{1-e^{\frac{-(V+52)}{5}}}+{0.5e}^{\frac{-(V+57)}{40}}}$$ (31)

$$r_{\infty }(V)=\frac{1}{1+e^{\frac{V+82.9}{12.4}}},\tau_{r}(V)=\frac{136.36e^{0.033(V+75)}}{1+e^{0.083(V+75)}}$$ (32)

The PYR received excitatory input from itself, with maximal conductance *g*_*ee*_, reversal potential *E*_*e*_, and synaptic variable *S*_*e*_; and inhibitory input from the INT, with maximal synaptic conductance *g*_*ei*_, reversal potential *E*_*i*_, and synaptic variable *S*_*i*_. The synaptic variables were modeled as in **Eqs 12–14**.

For the basic component of the INT we used the Wang-Buzsáki model [62] describing the dynamics of the membrane potential (*V*^*i*^), sodium inactivation (*h*), and delayed-rectifier potassium (*n*). To model gamma resonance in the INT (**Fig 8**), the model was extended to include a non-inactivating potassium current (*q*) with dynamics similar to but faster than an M-current [63]. The full model also included synaptic currents and noise, and reads

$$C\frac{dV^{i}}{dt}=I_{in}^{i}(t)-g_{L}^{i}(V^{i}-E_{L}^{i})-g_{Na}^{i}h{m_{\infty }(V^{i})}^{3}(V^{i}-E_{Na}^{i})-g_{K}^{i}n^{4}(V^{i}-E_{K}^{i})-g_{M}^{i}q(V^{i}-E_{K}^{i})-g_{ie}S_{e}(V^{e})(V^{i}-E_{e})-g_{ii}S_{i}(V^{i})(V^{i}-E_{i})+g_{N}\eta^{i}(t)$$ (33)

$$\frac{dh}{dt}=\frac{h_{\infty }(V^{i})-h}{\tau_{h}(V^{i})}$$ (34)

$$\frac{dn}{dt}=\frac{n_{\infty }(V^{i})-n}{\tau_{n}(V^{i})}$$ (35)

$$\frac{dq}{dt}=\frac{q_{\infty }(V^{i})-q}{\tau_{q}(V^{i})}$$ (36)

The gating variables for the INT (*x = h*,*m*,*n*,*q*) had voltage-dependent time constants (*τ*_*x*_) and steady-state values (*x*_*∞*_) as follows

$$h_{\infty }(V)=\frac{{0.07e}^{\frac{-(V+58)}{20}}}{{0.07e}^{\frac{-(V+58)}{20}}+\frac{1}{1+e^{\frac{-(V+28)}{10}}}},\tau_{h}(V)=\frac{0.2}{{0.07e}^{\frac{-(V+58)}{20}}+\frac{1}{1+e^{\frac{-(V+28)}{10}}}}$$ (37)

$$m_{\infty }(V)=\frac{\frac{0.2(V+35)}{1-e^{\frac{-(V+35)}{10}}}}{\frac{0.2(V+35)}{1-e^{\frac{-(V+35)}{10}}}+{4e}^{\frac{-(V+60)}{18}}}$$ (38)

$$n_{\infty }(V)=\frac{\frac{0.01(V+34)}{1-e^{\frac{-(V+34)}{10}}}}{\frac{0.01(V+34)}{1-e^{\frac{-(V+34)}{10}}}+{0.125e}^{\frac{-(V+44)}{80}}},\tau_{n}(V)=\frac{0.2}{\frac{0.01(V+34)}{1-e^{\frac{-(V+34)}{10}}}+{0.125e}^{\frac{-(V+44)}{80}}}$$ (39)

$$q_{\infty }(V)=\frac{1}{1+e^{\frac{-(V+35)}{10}}},q_{r}(V)=\frac{40}{3.3e^{\frac{V+35}{20}}+e^{\frac{-(V+35)}{10}}}$$ (40)

The INT received excitatory input from the PYR, with maximal synaptic conductance *g*_*ie*_; and inhibitory input from itself, with maximal synaptic conductance *g*_*ii*_.

For modeling the PYR in isolation (**Fig 7A**) or the γINT in isolation (**Fig 8AB**), all synaptic conductance values were set to zero. For modeling the INT-to-PYR network without gamma resonance on the INT (**Fig 7B**), *g*_*M*_^*i*^ was set to zero. The full model was used for **Fig 8C**. Specific parameter values followed [58], and are detailed in **Table 3**.

**Table 3:** Parameters used for modeling IPSP-induced network resonance (Figs 7–8).

Parameter	Value	Units	Notes
C^e^	1	μF/cm^2^	
g_L_^e^	0.1	mS/cm^2^	
E_L_^e^	-67	mV	
g_Na_^e^	100	mS/cm^2^	
E_Na_^e^	50	mV	
g_K_^e^	80	mS/cm^2^	
E_K_^e^	-100	mV	
g_h_^e^	0.485	mS/cm^2^	
E_h_^e^	-33	mV	
C^i^	1	μF/cm^2^	
g_L_^i^	0.1	mS/cm^2^	
E_L_^i^	-65	mV	
g_Na_^i^	35	mS/cm^2^	
E_Na_^i^	55	mV	
g_K_^i^	9	mS/cm^2^	
E_K_^i^	-90	mV	
g_M_^i^	4	mS/cm^2^	**Fig 7**: 0
τ_r_^e^	0.1	ms	AMPA
τ_d_^e^	3	ms	AMPA
E_e_	0	mV	AMPA
τ_r_^i^	0.3	ms	GABA_A_
τ_d_^i^	9	ms	GABA_A_
E_i_	-80	mV	GABA_A_
g_ie_	0	mS/cm^2^	PYR to INT
g_ee_	0	mS/cm^2^	PYR to PYR
g_ei_	0.4	mS/cm^2^	INT to PYR
g_ii_	0	mS/cm^2^	INT to INT
σ^e^	0.1	mV	
I_bias_^e^	-2.7	μA/cm^2^	
A_in_^e^	0	μA/cm^2^	**Fig 7A**: 0.2
σ^i^	0.1	mV	**Fig 8A**: 0
I_bias_^i^	-0.5	μA/cm^2^	**Fig 8AB and 8C**: 3.8, 3.7
A_in_^i^	0.5	μA/cm^2^	**Fig 8B and 8C**: 0.9, 2.1
